# Learning engagement and creative self-beliefs in generative AI-supported design education: self-reported prompt literacy and dimensions of AI dependence

**DOI:** 10.3389/fpsyg.2026.1957097

**Published:** 2026-10-28

**Authors:** Liduo Han, Zhongqin Cao

**Affiliations:** 1 School of Arts, Sichuan University Jinjiang College, Meishan, Sichuan, China; 2 Academy of Fine Arts, Aba Teachers University, Wenchuan, Sichuan, China

**Keywords:** AI dependence, creative personal identity, creative self-efficacy, design education, generative AI, learning engagement, self-reported prompt literacy

## Abstract

Generative AI (GenAI) is changing how design students learn, yet little is known about how students’ perceived capability to work with GenAI relates to their learning engagement and motivational self-beliefs, or under which forms of AI dependence this link is weaker. Drawing on social cognitive theory, this cross-sectional study examined self-reported prompt literacy, learning engagement, and two components of the creative self: creative self-efficacy (CSE), a motivational belief in one’s creative capability, and creative personal identity (CPI), the importance of creativity to one’s self-concept. Survey data came from 829 undergraduate art and design students in 40 intact classes at two institutions in mainland China; latent-variable and moderation models accounted for the clustering of students within classes. Self-reported prompt literacy was positively associated with engagement (*β* = 0.54), and engagement was positively associated with both CSE (*β* = 0.62) and CPI (*β* = 0.47). Engagement was more strongly associated with CSE than with CPI (difference = 0.14, 95% CI [0.04, 0.24]) and remained uniquely associated with CPI after CSE was taken into account. When four dimensions of AI dependence were modeled simultaneously, the prompt literacy–engagement association was weaker among students reporting higher cognitive dependence; evidence for loss of control was weaker, and functional and emotional dependence showed no clear unique moderation. All interaction effects were small. The dimensional analysis identified cognitive dependence as the clearest moderator. These cross-sectional, self-reported findings suggest possible boundary conditions for the capability–engagement association and require longitudinal and behavioral testing.

## 1 Introduction

GenAI now takes part in the core activities of design courses. Students use text-to-image and conversational systems to explore concepts, generate visual alternatives, and refine their work, and graphic-design educators describe a shift in students’ roles from executing designs toward curating and selecting AI-mediated outputs (Hwang and Wu, 2025a). Whether this supports learning depends less on access to the tools than on how students engage with them: a large-scale survey and a meta-analysis of university students found that associations between GenAI use and learning engagement varied with course design, teacher support, and the engagement dimension considered (Guo et al., 2025; Wang and Guo, 2025). Evidence on creative outcomes is similarly mixed. Studies of product-design ideation, art education, and short-story writing report gains in novelty, exploration, or rated quality alongside concerns about originality, foundational skills, and the diversity of outputs (Bartlett and Camba, 2024; Doshi and Hauser, 2024; Huang et al., 2024; Sáez-Velasco et al., 2024; Jiang et al., 2025), and a systematic review concludes that the consequences of human–AI co-creation vary by context and outcome (Heigl, 2026). Much of this evidence comes from populations adjacent to ours, such as graduate architecture students, product-design classes, online writers, and general university samples (Yaşar et al., 2025), so its relevance to undergraduate art and design students needs to be examined. This study focuses on how students’ perceived prompting capability relates to engagement and how engagement relates to their creative self-beliefs.

Students’ capability to work with GenAI has been described at several levels of generality. AI literacy commonly encompasses understanding, using, evaluating, and ethically applying AI, although existing scales do not share a single dimensional structure (Carolus et al., 2023; Wang et al., 2023), and it co-varies with related self-beliefs: in one cross-sectional study, AI literacy and AI self-efficacy both statistically mediated the association between attitudes toward AI and GenAI acceptance (Türk et al., 2025). GenAI literacy narrows the scope to generative systems; a workplace GenAI-literacy scale, for example, covers basic competence, prompt optimization, content evaluation, innovative application, and ethical compliance (Liu X. et al., 2025). Prompt literacy is narrower still. Tour and Zadorozhnyy (2025) define it as the knowledge, skills, and understanding needed for dynamic and iterative interaction with GenAI, organized around three steps: crafting a prompt, engaging with the response, and reviewing the prompt. Prompting competence, finally, refers to demonstrated performance, judged from the quality of prompts or of the outputs they produce; in a teaching study with six graduate architecture students, for instance, prompt formulation, professional vocabulary, and iteration were associated with the quality of generated work (Yaşar et al., 2025). The present study measures prompt literacy through students’ reports of these three steps in design tasks. We therefore refer to it as self-reported prompt literacy (PL) and interpret it as perceived prompting capability rather than demonstrated competence. Because AI self-efficacy and general digital confidence were not measured, the distinctiveness of PL from these self-beliefs remains untested.

Learning engagement (LE), students’ sustained behavioral, emotional, and cognitive involvement in learning activities (Fredricks et al., 2004; Kahu, 2013), is the process through which such capability is most likely to matter for learning. In creative disciplines, engagement may also bear on how students see themselves as creators. Research on the creative self distinguishes two related but distinguishable components (Karwowski, 2012; Karwowski et al., 2018): creative self-efficacy (CSE), the belief that one can produce creative outcomes (Tierney and Farmer, 2002), and creative personal identity (CPI), the extent to which creativity is an important part of one’s self-concept. CSE is a motivational self-belief in the social cognitive sense, because beliefs about one’s capabilities shape the goals people choose, the effort they invest, and how long they persist (Bandura, 1997). CPI concerns what students value and how they define themselves. Two recent studies of university students linked AI literacy to these beliefs, but one placed identity downstream of efficacy (Li H. et al., 2026), and the other combined the two beliefs into a single composite and located the intervening process in problem-solving ability (Peng et al., 2026). Neither examined learning engagement, and neither could show whether engagement is related equally to the belief that one can create and to the sense that creativity is part of who one is. This is the principal gap the present study addresses.

A secondary gap concerns the link on which this question builds: the conditions under which perceived prompting capability is less closely tied to engagement. AI dependence (AID) is one such condition, and it is increasingly treated as multidimensional: available instruments distinguish trust in AI, informational exclusivity with AI, and AI literacy (Garcia Castro et al., 2025); reflective, cautious, thoughtless, and collaborative reliance during problem solving (Hou et al., 2025); and emotional dependence, functional dependence, cognitive dependence, and loss of control (Wu et al., 2026). Survey studies of university students link GenAI use to both task self-efficacy and technological dependence (Zhang and Xu, 2025), and link dependence to cognitive inertia and lower innovative capability (Yang et al., 2025), to weaker critical thinking (Tian and Zhang, 2026), and to academic stress, burnout, and anxiety (Liu et al., 2026; Wang et al., 2026). Another study separated AI dependence from reflective collaboration and related the two patterns to psychological ownership and competency anxiety (Li X. et al., 2026), and an opinion article has warned of cognitive erosion without testing it empirically (Jose et al., 2025). Because these studies are cross-sectional, they show which forms of dependence co-occur with less favorable outcomes rather than what dependence does. What remains unclear is which dimensions, considered together, mark a weaker association between students’ prompting capability and their engagement, a question that a total dependence score cannot answer.

The present study addresses these gaps among undergraduate art and design students in blended, GenAI-supported courses at two institutions. It asks how self-reported prompt literacy relates to learning engagement, whether engagement is associated equally with CSE and CPI, and which AI-dependence dimensions mark conditions under which the prompt literacy–engagement association is weaker. The study contributes to research on GenAI, motivation, and engagement by examining CSE and CPI separately and by comparing the four dependence dimensions in a joint model. These comparisons allow us to ask whether engagement relates differently to creative efficacy and identity, and whether particular forms of dependence are associated with a weaker capability–engagement link. Because the data are cross-sectional and self-reported, all analyses estimate concurrent associations within one theoretically motivated specification; they do not establish temporal order, causal effects, or mediating mechanisms (Maxwell and Cole, 2007; Rohrer et al., 2022).

### 1.1 Theoretical framework and hypotheses

Social cognitive theory explains human functioning through interacting personal, behavioral, and environmental factors and emphasizes people’s capacity to exercise agency (Bandura, 1986, 2001). GenAI adds a distinctive element to the learning environment: a system that can act on the learner’s behalf. Relying on it resembles what Bandura calls proxy agency, in which others with the requisite expertise act on one’s behalf; people sometimes turn to proxy agency because they do not want to take on the arduous work of developing the necessary competencies themselves (Bandura, 2000). This perspective informs each relationship examined here. Perceived capability to direct GenAI, by formulating and refining prompts and evaluating outputs, is a personal factor that should relate to students’ choice of, and persistence in, learning activities (H1). Engagement in course tasks supplies enactive mastery experiences, the most influential source of efficacy beliefs (Bandura, 1997), and self-relevant experiences from which students may come to regard creativity as part of who they are (H2a, H2b). Dependence that substitutes AI for students’ own thinking, or that reflects difficulty regulating AI use, leaves less room for personal agency and should therefore weaken the link between perceived prompting capability and engagement (H4a, H4b). The theory does not prescribe a single ordering of these factors; reciprocal influences are expected (Bandura, 1986). We therefore test one theoretically motivated ordering of concurrent associations, not a causal chain.

Self-reported prompt literacy should be positively associated with engagement because GenAI-supported design tasks reward the ability to direct the system. Students who feel able to frame prompts around a design goal, evaluate outputs, and revise their requests may be more willing to persist with a task. Evidence from general student samples is consistent with this expectation: a large-scale survey and a meta-analysis found GenAI use to be positively associated with engagement on average (Guo et al., 2025; Wang and Guo, 2025), and a survey found information literacy to be positively associated with online learning engagement (Zhao, 2024). Because this evidence is correlational and comes from other populations, it supports a directional expectation rather than a causal claim. In this study, engagement refers to involvement in course learning, not to the amount of interaction with AI. These considerations led to H1:

*H1*: Prompt literacy is positively associated with learning engagement (PL → LE).

Engagement should be positively associated with CSE. In social cognitive theory, enactive mastery experiences are the most influential source of efficacy beliefs (Bandura, 1997), and engagement in design tasks, such as comparing options, interpreting outputs, discussing work, and revising it, is where such experiences arise. Creativity research shows that CSE relates to creative performance (Tierney and Farmer, 2002), and studies in adjacent populations place CSE and engagement within a common set of motivational relationships: CSE is linked to school climate, creativity motivation, and creative performance (He and Zhang, 2025) and to problem-solving skills among engineering students (Liu Z. et al., 2025); among design students, GenAI self-efficacy, anxiety, and creative cognition are related (Hwang and Wu, 2025b); and engagement lies on statistical pathways from motivation to creative idea generation in online learning (Wang, 2022) and to research innovation among master’s students (Hu et al., 2025). This reasoning led to H2a:

*H2a*: Learning engagement is positively associated with creative self-efficacy (LE → CSE).

Engagement should also be positively associated with CPI, the extent to which individuals regard creativity as an important part of their self-concept (Karwowski, 2012; Karwowski et al., 2018). CPI is related to CSE but answers a different question: not what students can do, but who they take themselves to be. Two lines of theory connect it with engagement. Self-perception theory holds that people infer their own attributes partly from their behavior (Bem, 1972), and identity research ties the activities people pursue and value to how they define themselves (Eccles, 2009). Sustained engagement in design work gives students repeated, self-relevant creative experiences from which they may infer that creativity is part of who they are. Consistent with this, Li H. et al. (2026) found positive associations of AI literacy with both CSE and CPI among university students, although their cross-sectional data do not establish that efficacy precedes identity. We therefore proposed H2b:

*H2b*: Learning engagement is positively associated with creative personal identity (LE → CPI).

Whether engagement is related equally to the two beliefs is less clear. Social cognitive theory suggests one reason to expect a closer link with efficacy: mastery experiences bear most directly on beliefs about what one can do (Bandura, 1997), whereas identity also rests on the value people attach to an activity and on longer-term self-definition (Eccles, 2009). This reasoning concerns the sources of the two beliefs, however. We are not aware of studies that have compared their associations with learning engagement, and the conceptual distinction between CSE and CPI does not by itself establish which association should be stronger. We therefore state the comparison as a research question:

RQ1: Is learning engagement more strongly associated with creative self-efficacy than with creative personal identity?

Self-reported prompt literacy may also be related to the creative self in ways not captured by engagement, for example because students who rate their prompting capability highly may rate their creative capability highly as well. The model therefore retains direct paths from PL to CSE and CPI. These paths are part of the model specification rather than hypotheses: fixing them to zero would impose complete transmission through engagement, a strong restriction for which neither theory nor prior evidence provides a basis, and would bias the engagement paths if PL is related to the creative self in other ways. Studies of online creative learning and of research innovation have reported statistical indirect roles for engagement (Wang, 2022; Hu et al., 2025). Because those studies, like ours, are observational, we use the term indirect association rather than mediating mechanism. The difference between the two indirect associations shares the PL → LE path and is therefore examined as part of RQ1. This yielded the following hypotheses:

*H3a*: Prompt literacy is indirectly associated with creative self-efficacy through learning engagement (PL → LE → CSE).

*H3b*: Prompt literacy is indirectly associated with creative personal identity through learning engagement (PL → LE → CPI).

The four AIDep-22 dimensions describe different forms of reliance (Wu et al., 2026). Cognitive dependence refers to letting AI think and judge in one’s place, for example asking for solutions before attempting one’s own reasoning; loss of control refers to difficulty regulating AI use, such as failed attempts to cut down, difficulty stopping, and urges to use; functional dependence refers to delegating tasks to AI for efficiency; and emotional dependence refers to feeling secure when AI is available and uneasy without it. From a social cognitive perspective, cognitive dependence and loss of control are the forms most clearly opposed to personal agency. Students who habitually substitute AI output for their own judgment are more likely to use their prompting capability to obtain ready-made answers than to explore, compare, and revise, so the association of that capability with engagement should be weaker among them (H4a). Self-regulation is the process through which people direct their behavior toward chosen goals (Bandura, 1991), and research on media use treats deficient self-regulation as a source of habitual, unregulated use (LaRose et al., 2003). Students who struggle to regulate their AI use may therefore channel their prompting capability less consistently into course learning (H4b). This expectation concerns the quality of regulation, not the quantity of use: the loss-of-control items describe failed attempts to control use, including its frequency and duration, rather than how often AI is used, and the engagement measure concerns involvement in course learning rather than interaction with AI. Frequent use could accompany either high or low engagement, so weekly use frequency is treated as a covariate rather than as an indicator of dependence. Functional delegation and the comfort of having AI available do not in themselves imply that students stop thinking, and evidence on whether they alter engagement is limited, so no directional prediction was made for these two dimensions. Consistent with this distinction, a survey of Chinese university students found cognitive dependence to be more strongly associated with cognitive inertia than tool dependence (Yang et al., 2025). The study therefore enters all four dimensions simultaneously and tests the following hypotheses of differential moderation:

*H4a*: Cognitive dependence negatively moderates the association between prompt literacy and learning engagement.

*H4b*: Loss of control negatively moderates the association between prompt literacy and learning engagement.

*H4c*: The negative moderating effects of cognitive dependence and loss of control are stronger than those of functional and emotional dependence (moderation-difference hypothesis).

The corresponding conditional indirect associations are exploratory, are reported in the Supplementary material, and were not assigned a separate hypothesis. All moderation hypotheses were specified before analysis.

The study tests H1 (PL → LE), H2a and H2b (LE → CSE and LE → CPI), H3a and H3b (indirect associations through engagement), and H4a–H4c (differential moderation across dependence dimensions), and addresses RQ1 (the difference between the LE → CSE and LE → CPI associations) among undergraduate art and design students at Institution A and Institution B. Figure 1 presents this main model (M1). Direct paths from PL to CSE and CPI are part of its specification. A restricted efficacy-gateway model (M2; PL → LE → CSE → CPI) serves as a structural sensitivity analysis.

**Figure 1:**
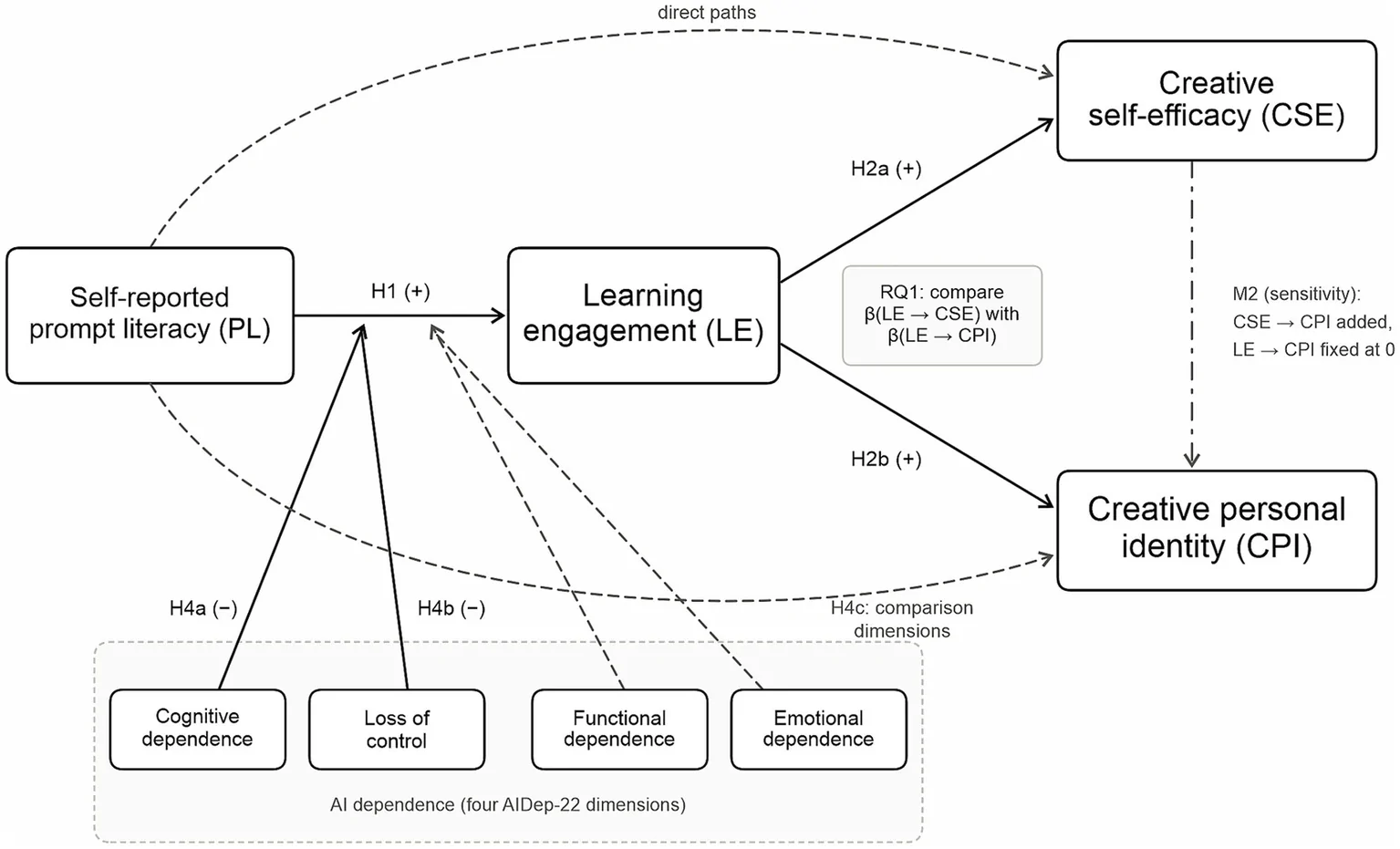
Hypothesized model. Solid arrows show H1, H2a, and H2b; dashed curves show the direct paths from PL. The dash-dotted arrow is the CSE → CPI path of the restricted model (M2), in which LE → CPI is fixed at zero. Among the moderators, solid arrows mark H4a and H4b and dashed arrows the comparison dimensions (H4c). Main effects of the dependence dimensions on LE are not drawn.

## 2 Materials and methods

### 2.1 Study design, participants, and sampling

This cross-sectional survey drew on undergraduate art and design students with experience of GenAI-supported design learning — AI-assisted information retrieval, visual concept generation, style exploration, and iterative refinement in blended and flipped design courses. Participants came from two institutions: Institution A, the main site, covered second- to fourth-year students in five design specialisms; Institution B covered second- and third-year students in three. Because each sector was represented by a single institution, institution and sector are fully confounded, and we therefore use the neutral labels Institution A and Institution B throughout. Questionnaires were administered within intact classes via Wenjuanxing; class identifiers were recorded only to adjust for clustering, as stated in the consent form. Data were collected in June 2026.

A total of 863 questionnaires were returned. Thirty-four were removed using the prespecified sequence of eligibility screening, attention checks, completion time, straight-lining, and duplicate IP addresses: three respondents failed the eligibility item and the survey ended automatically; 16 failed an attention check (ATT1 ≠ 2 or ATT2 ≠ 6); six completed the survey in less than 180 s; five selected the same response for all 58 items; and four were duplicate submissions from the same IP address, for which the first submission was retained. The final sample comprised 829 valid responses (96.1%): 573 students in 28 classes at Institution A and 256 students in 12 classes at Institution B, for a total of 40 classes. Class size ranged from 17 to 24 students (*M* = 20.7). Participants had a mean age of 20.86 years (SD = 0.92; range 19–24). The 829 valid cases had complete responses on all 58 core items, so no imputation was required. Sample characteristics are reported in Table 1.

**Table 1:** Demographic profile of the valid sample by institution (Institution A/Institution B).

Demographic	Category	Institution A *n* (%)	Institution B *n* (%)	Total *n* (%)
Age *M* (SD)	—	20.99 (0.97)	20.57 (0.72)	20.86 (0.92)
Sex	Female	321 (56.0%)	140 (54.7%)	461 (55.6%)
	Male	252 (44.0%)	116 (45.3%)	368 (44.4%)
Year of study	Second year	218 (38.0%)	129 (50.4%)	347 (41.9%)
	Third year	186 (32.5%)	127 (49.6%)	313 (37.8%)
	Fourth year	169 (29.5%)	0 (0.0%)	169 (20.4%)
Design specialism	Visual communication	183 (31.9%)	83 (32.4%)	266 (32.1%)
	Digital media art	141 (24.6%)	87 (34.0%)	228 (27.5%)
	Environmental/interior design	127 (22.2%)	86 (33.6%)	213 (25.7%)
	Product design	79 (13.8%)	0 (0.0%)	79 (9.5%)
	Other	43 (7.5%)	0 (0.0%)	43 (5.2%)
Weekly GenAI use	<1 times	17 (3.0%)	23 (9.0%)	40 (4.8%)
	1–3 times	220 (38.4%)	83 (32.4%)	303 (36.6%)
	4–6 times	213 (37.2%)	91 (35.5%)	304 (36.7%)
	Daily	123 (21.5%)	59 (23.0%)	182 (22.0%)
Number of classes		28	12	40
Valid sample size		573	256	829

Institution B did not include fourth-year students or product/other design specialisms.

Post-survey intraclass correlation coefficients, ICC(1), for the core constructs, using the 40 instructional classes as clusters, were low: PL = 0.028 (design effect = 1.54), LE = 0.041 (1.81), AID = 0.000 (1.00), CSE = −0.003 (0.94), and CPI = 0.006 (1.11). Low ICCs, however, do not mean that clustering can be ignored; conventional cluster-robust inference itself requires caution when the number of clusters is limited (Pustejovsky and Tipton, 2018). Instead, the main M1 paths, path difference, indirect associations, and *R*^2^ values were estimated through 2,000 bootstrap resamples of the 40 intact classes, and the moderation analyses used the same class-level bootstrap. Institution was included as a fixed covariate in the adjusted sensitivity models because only two institutions were sampled.

### 2.2 Procedure and ethics

The study was conducted in accordance with the Declaration of Helsinki. The study was approved by the Ethics Committee of Sichuan University Jinjiang College (approval no. SCJJKJL-2026-007). Before completing the survey, all participants read an information sheet describing the study purpose, voluntary participation, anonymity, the exclusive statistical use of class information, and the scope of data use. All participants provided online informed consent by confirming their agreement before entering the questionnaire; respondents who did not confirm could not proceed to the items. All participants were adults (*M* = 20.86 years, range 19–24). They could withdraw at any time without academic consequences. The Wenjuanxing survey was administered within intact classes, contained two attention checks placed in the learning-engagement and AI-dependence sections, and had a median completion time of 8.9 min.

### 2.3 Measures

All core variables were measured on seven-point Likert scales. Source instruments with five response options were converted to seven points so that respondents answered all 58 items in a single format. English-language items were translated into Chinese and independently back-translated, with discrepancies resolved through discussion. Two bilingual experts reviewed all items for conceptual equivalence and clarity in blended design learning, and cognitive interviews with eight students outside the pilot and main samples used think-aloud and verbal-probing procedures to identify divergent interpretations. Because a changed response format, translation, and contextual adaptation can each alter an instrument’s psychometric properties, we evaluated the adapted measures in the present sample: response distributions, reliability, item- and parcel-level factor structure, discriminant validity, and measurement invariance are reported in the Results. Comparisons of response formats provide some support for using seven response categories (Preston and Colman, 2000), but this general evidence is secondary to the present-sample results. Raw means are not directly comparable with studies that used the original five-point formats.

#### 2.3.1 Self-reported prompt literacy (PL)

The study used a six-item self-report indicator drawn mainly from items on prompt optimization and generated-content evaluation in the workplace GenAI-literacy scale developed by Liu X. et al. (2025). Wording was adapted to design learning with reference to the conceptualization by Tour and Zadorozhnyy (2025) and the prompting characteristics observed by Yaşar et al. (2025) in architectural-design tasks. The six items correspond to the three interaction steps in that conceptualization: crafting prompts (PL1–PL2), reviewing and revising prompts (PL3), and engaging critically with generated responses (PL4–PL6); full item wording appears in Supplementary Table S6. Because the source instrument was not an independent prompt-literacy scale for design education and the present measure captures students’ self-perceptions, the variable should be interpreted as contextualized self-reported prompt-related capability rather than objective prompting performance. Cronbach’s alpha was 0.829 in the present sample.

#### 2.3.2 Learning engagement (LE)

The 19-item measure was adapted from Dixson’s Online Student Engagement Scale (OSE) (Dixson, 2015), which comprises skills, emotional, participation, and performance engagement. Four of the 19 items were respecified to design coursework (for example, “making sure to study on a regular basis” became “making sure to study design course content on a regular basis”). Because all participating courses used blended or flipped formats, the instructions directed students to the online-platform components of their courses. Cronbach’s alpha was 0.907 in the present sample.

#### 2.3.3 AI dependence (AID)

The 22-item measure was adapted from Wu et al.’s AIDep-22 (Wu et al., 2026) and covered emotional dependence, functional dependence, cognitive dependence, and loss of control. Because AIDep-22 was developed and validated with Chinese undergraduate samples, no translation was required; its original Chinese items were retained, with references to academic tasks respecified as design tasks in 11 of the 22 items. Cronbach’s alpha was 0.900 in this sample.

#### 2.3.4 Creative self (CSE and CPI)

The Short Scale of Creative Self (SSCS; Karwowski, 2012; Karwowski et al., 2018) was used, comprising six creative-self-efficacy items and five creative-personal-identity items. The instructions asked students to respond with reference to their overall experience of design learning, but the items themselves were not deeply rewritten for the context. Cronbach’s alpha was 0.846 for CSE and 0.807 for CPI in the present sample.

#### 2.3.5 Pilot study

The complete instrument was piloted in an independent sample. Of 110 returned questionnaires, 104 were retained after excluding five failed attention checks and one patterned response. Across the five scales, item-discrimination statistics ranged from 5.89 to 13.89 (all *p* < 0.001), corrected item–total correlations from 0.44 to 0.78, and Cronbach’s alpha from 0.812 to 0.935 (Supplementary Table S1A). Principal-axis factoring with Promax rotation of the 11 translated creative-self items and six adapted prompt-literacy items retained the expected three-factor structure, accounting for 60.0% of the variance and supported by parallel analysis with 1,000 replications. All items loaded primarily on their intended factor; the largest cross-loading was 0.225. AI dependence and learning engagement proceeded directly to confirmatory factor analysis (CFA) in the main sample because AIDep-22 was developed with Chinese undergraduates and the OSE has an established four-dimension structure. The pilot and main samples did not overlap. Full pilot results and factor matrices are reported in Supplementary Table S1; item-level CFA and heterotrait–monotrait ratio (HTMT) results from the main sample appear in Supplementary Table S2.

### 2.4 Data analysis

The analysis followed a two-stage approach in which the measurement model preceded the structural model (Anderson and Gerbing, 1988). First, a correlated 11-factor CFA was estimated from the 58 items. Model fit was described with the comparative fit index (CFI), the Tucker–Lewis index (TLI), the root mean square error of approximation (RMSEA), and the standardized root mean square residual (SRMR). For the structural models, learning engagement and AI dependence were each represented by a single latent factor indicated by the means of their four dimensions (Little et al., 2013), whereas PL, CSE, and CPI were indicated by their items. Parceling kept the content of every dimension within each construct while reducing the number of free parameters relative to the 40 class clusters; because parcels can conceal item-level misspecification (Marsh et al., 2013), the item-level 11-factor model is reported alongside the parcel-based model. The main model, M1, was a parallel dual-outcome structural equation model (PL → LE → CSE/CPI) that retained the direct PL → CSE and PL → CPI paths and freely estimated the CSE–CPI residual covariance. Its structural part is just-identified, so its fit equals that of the corresponding measurement model and cannot by itself support the hypothesized ordering. RQ1 was evaluated through the difference between the standardized LE → CSE and LE → CPI paths and the corresponding difference between the two indirect associations. The restricted model M2 removed LE → CPI and replaced the CSE–CPI residual covariance with a CSE → CPI path. Replacing the residual covariance with a CSE → CPI path while retaining LE → CPI yields an equivalent model with identical fit, which we label M1′ (Lee and Hershberger, 1990; MacCallum et al., 1993). M2 is nested in M1′ and differs from it only by the constraint LE → CPI = 0. This restriction was tested using a scaled likelihood-ratio test with 1 df (Satorra and Bentler, 2001). Because M1, M1′, and a model with CPI → CSE are equivalent, this test cannot identify the ordering of CSE and CPI; it asks only whether engagement retains a unique association with CPI once CSE is taken into account. Because 829 students were nested within 40 classes, uncertainty for the core M1 results was estimated with a class-level bootstrap with 2,000 resamples and bias-corrected (BC) intervals. Under TYPE = COMPLEX, Mplus draws K − 1 = 39 classes with replacement in each resample, retains all students in the selected classes, and rescales the weights (Asparouhov and Muthén, 2010). An individual-level bootstrap was used only as a sensitivity comparison. A Wald test of the equality of the two engagement paths is reported only as descriptive evidence, because it relies on the cluster-robust information matrix, which is imprecise with 40 clusters.

The primary moderation model entered PL, all four dependence dimensions, and their four interaction terms simultaneously as predictors of learning engagement. H4c contrasted the summed cognitive-dependence and loss-of-control interactions with the summed functional- and emotional-dependence interactions. Interaction terms were constructed from standardized composite scores. Simple slopes were derived from this model with the other dependence dimensions and their interactions fixed at zero; conditional indirect associations and indices of moderated mediation (Hayes, 2015) were derived in the same way but are exploratory. Coefficients, contrasts, and conditional effects were estimated with 2,000 class-level bootstrap resamples and BC 95% confidence intervals. Because inference based on few clusters can be unreliable (Pustejovsky and Tipton, 2018), the key coefficients of the moderation model and of a composite-score version of M1 were also tested with bias-reduced (CR2) cluster-robust standard errors and Satterthwaite degrees of freedom, and leave-one-class-out analyses re-estimated M1 and the moderation model 40 times, each time omitting one class. In covariate-adjusted models, institution, sex, year of study, design specialism, and weekly GenAI use were entered as dummy-coded variables. Descriptive statistics, reliability estimates, HTMT ratios, intraclass correlations, variance inflation factors (VIFs), and Harman’s single-factor test were computed in IBM SPSS Statistics 27. CFA, structural, measurement-invariance, and moderation models were estimated in Mplus 8.3 with TYPE = COMPLEX and CLUSTER = class; maximum likelihood with robust standard errors (MLR) was used for model fit and information criteria. Because M1 contained more free parameters (69) than clusters (40), Mplus warned that its cluster-robust (sandwich) standard errors might not be trustworthy; inferential tests for M1 and the moderation model therefore used the class-level bootstrap, and all 2,000 resamples converged. The CR2 tests, the leave-one-class-out analyses, the covariate-adjusted models, the equivalent model M1′, and the constrained discriminant-validity model were run in R 4.3.3 (R Core Team, 2024) with lavaan 0.6.17 (Rosseel, 2012) and clubSandwich 0.5.10 (Pustejovsky, 2023); for M1, the lavaan path estimates matched the Mplus estimates to three decimals.

Analyses were organized in three tiers. Confirmatory analyses tested H1, H2a, H2b, H3a, H3b, and H4a–H4c in M1 and the simultaneous moderation model; RQ1 was addressed in M1 as a comparison without a directional prediction. Sensitivity analyses examined whether these results depended on particular analytic choices: the M2 restriction test, covariate adjustment, institution-specific estimates, re-estimation without performance engagement, composite-score models, individual- versus class-level resampling, CR2 tests, and leave-one-class-out estimates. Exploratory analyses, namely total-score and single-dimension moderation models, pairwise contrasts among dimensions, and conditional indirect associations, were not used to test the hypotheses; they are reported briefly in the text and in detail in the Supplementary material.

## 3 Results

### 3.1 Common method bias

In Harman’s single-factor test, the first factor explained 18.8% of the variance. A single-factor CFA of all 58 items fit poorly [χ^2^(1595) = 11215.98, CFI = 0.431, TLI = 0.411, RMSEA = 0.085] and was clearly inferior to the 11-factor model. After adding an unmeasured method factor with equal loadings, the method factor accounted on average for 3.6% of indicator variance (standardized loadings 0.168–0.219), the improvement in fit was negligible [scaled Δχ^2^(1) = 1.00, *p* = 0.32], and the primary loading pattern remained stable. These diagnostics argue against a single dominant response factor, but they detect shared method variance poorly and cannot rule it out (Podsakoff et al., 2003). Because all variables came from the same respondents, the associations among the self-reported constructs may be partly inflated by common method variance.

### 3.2 Measurement model: reliability and validity

The correlated 11-factor item-level CFA showed acceptable fit [χ^2^(1540) = 2393.66, *p* < 0.001, CFI = 0.950, TLI = 0.946, RMSEA = 0.026, SRMR = 0.033]; all 58 standardized item loadings ranged from 0.546 to 0.794 and were significant at *p* < 0.001. Responses used the full seven-point range: all seven categories were used for 56 of the 58 items (no respondent chose 1 for two prompt-literacy items), item standard deviations ranged from 1.14 to 1.49, item skewness ranged from −0.56 to 0.33, and no item had more than 25.8% of its responses at either end of the scale. The five-factor parcel-based measurement model (LE and AID each indicated by their four dimension means; PL, CSE, and CPI by their items) also fitted well (χ^2^(265) = 483.80, *p* < 0.001, CFI = 0.970, TLI = 0.966, RMSEA = 0.032, 90% CI [0.027, 0.036], SRMR = 0.033). Mardia’s test based on the 21 structural-model indicators indicated significant multivariate skewness and kurtosis (skewness χ^2^ = 2338.3, *p* < 0.001; kurtosis z = 10.41, *p* < 0.001). Model fit was therefore evaluated with the Satorra–Bentler scaled statistic produced by MLR (scaling correction factor 1.077 for M1) together with approximate fit indices, and inference for all key effects rested on bias-corrected bootstrap confidence intervals robust to non-normality.

Table 2 reports descriptive statistics, Cronbach’s alpha, composite reliability (CR), average variance extracted (AVE), and standardized loading ranges for the five constructs; results for the first-order dimensions appear in Supplementary Table S2A. CR values for the five constructs ranged from 0.768 to 0.847, and all loadings were significant and at least 0.546. AVE ranged from 0.452 to 0.544 and exceeded 0.50 only for LE; the values for PL (0.452), AID (0.453), CSE (0.480), and CPI (0.457) fell below it. Reliability was therefore adequate (CR ≥ 0.768), but for these four constructs the indicators shared less than half of their variance with the construct, so convergent validity is weaker than reliability (Fornell and Larcker, 1981). Latent-variable models account for measurement error under the specified measurement model, but this does not resolve concerns about item content or model misspecification. The low AVE values therefore qualify the interpretation of the structural estimates as well as their precision. The overlap between LE and CSE is an additional concern for the comparison addressed by RQ1. Possible reasons for the low AVE, which differ across constructs, are considered in the Limitations. Latent correlations among the four AI-dependence dimensions ranged from 0.52 to 0.58, with each dimension retaining unique variance, providing an empirical basis for dimension-specific tests.

**Table 2:** Descriptive statistics, reliability, and convergent validity of the five constructs (*N* = 829).

Construct	Items	*M*	SD	α	CR	AVE	Std. loading range
Self-reported prompt literacy, PL	6	5.17	0.94	0.829	0.831	0.452	0.583–0.757
Learning engagement, LE (parcel indicators)	19	5.08	0.80	0.907	0.827	0.544	0.683–0.769
AI dependence, AID (parcel indicators)	22	3.61	0.78	0.900	0.768	0.453	0.645–0.708
Creative self-efficacy, CSE	6	5.00	1.02	0.846	0.847	0.480	0.662–0.739
Creative personal identity, CPI	5	4.70	0.97	0.807	0.806	0.457	0.603–0.793

For LE and AID, α is based on the items (19 and 22); CR, AVE, and loadings refer to their four dimension-mean parcels in the five-factor CFA.

Table 3 reports the discriminant-validity evidence. Latent correlations among the five constructs ranged from 0.061 to 0.705. One pair was borderline: the LE–CSE correlation (0.705) exceeded the square root of the AVE of CSE (0.693), so these two constructs did not meet the Fornell–Larcker criterion. Three further indicators bear on this pair. Their HTMT ratio was 0.705, below the 0.85 threshold (Henseler et al., 2015); the 95% confidence interval of their latent correlation was [0.656, 0.754], with an upper limit below 0.80, a level at which correlations are not usually taken to indicate a discriminant-validity problem (Rönkkö and Cho, 2022); and constraining the correlation to 0.85 worsened model fit significantly [scaled Δχ^2^(1) = 49.8, *p* < 0.001]. LE and CSE are therefore strongly related but empirically separable. Because their overlap is the largest in the model, it matters most for RQ1; the robustness of that comparison across specifications is reported under Robustness checks. The HTMT ratio for CSE and CPI was 0.619 and their latent correlation 0.635, consistent with two related but distinguishable components of the creative self. Latent correlations among the engagement dimensions were 0.68–0.75, consistent with a common engagement factor (Little et al., 2013).

**Table 3:** Discriminant validity of the five constructs: Fornell–Larcker criterion and HTMT.

Construct	PL	LE	AID	CSE	CPI
PL	(0.672)	0.549	0.160	0.506	0.452
LE	0.539	(0.738)	0.061	0.705	0.570
AID	0.165	0.061	(0.673)	0.082	0.132
CSE	0.495	0.705	0.078	(0.693)	0.619
CPI	0.440	0.574	0.131	0.635	(0.676)

Diagonal, square root of AVE; below the diagonal, latent correlations from the five-factor CFA; above, HTMT. The 95% CI of the LE–CSE latent correlation is [0.656, 0.754].

### 3.3 Descriptive statistics and correlations

Zero-order correlations among the composite scores were broadly consistent with the main model (Table 4). Prompt literacy was positively correlated with learning engagement (*r* = 0.447), CSE (*r* = 0.423), and CPI (*r* = 0.368); learning engagement was also positively correlated with CSE (*r* = 0.585) and CPI (*r* = 0.464). Zero-order correlations of the total AI-dependence score and its four dimensions with engagement were weak and nonsignificant (absolute r at or below 0.044), and their correlations with prompt literacy were also weak (*r* at or below 0.142). Zero-order correlations with demographic variables appear in Supplementary Table S3. These near-zero marginal correlations do not demonstrate that AI dependence operates mainly through moderation. They show only that no clear linear association was observed before accounting for shared variance among the dimensions and for interaction terms.

**Table 4:** Descriptive statistics and correlations of composite scores (*N* = 829).

Variable	*M*	SD	1	2	3	4	5	6	7	8	9	Skewness	Kurtosis
1. Self-reported prompt literacy, PL	5.17	0.94	—									−0.31	−0.25
2. Learning engagement, LE	5.08	0.80	0.447***	—								−0.33	−0.10
3. Total AI-dependence score	3.61	0.78	0.126***	0.044	—							0.30	0.46
4. Emotional dependence	3.66	0.97	0.112**	0.044	0.760***	—						0.22	−0.06
5. Functional dependence	3.76	1.05	0.099**	0.029	0.793***	0.498***	—					0.21	−0.12
6. Cognitive dependence	3.39	1.03	0.142***	0.038	0.741***	0.442***	0.434***	—				0.16	−0.24
7. Loss of control	3.59	0.99	0.042	0.028	0.772***	0.459***	0.439***	0.442***	—			0.14	−0.04
8. Creative self-efficacy, CSE	5.00	1.02	0.423***	0.585***	0.061	0.071*	0.019	0.072*	0.035	—		−0.36	−0.11
9. Creative personal identity, CPI	4.70	0.97	0.368***	0.464***	0.101**	0.101**	0.061	0.081*	0.072*	0.514***	—	−0.22	−0.18

Composite scores are item means. *p < 0.05, **p < 0.01, ***p < 0.001.

### 3.4 Measurement invariance across institutions

Cross-institutional measurement-invariance testing (Institution A, *n* = 573; Institution B, *n* = 256) showed good fit for the configural model (Table 5). Imposing equal-loadings constraints produced little change in fit (metric invariance: Δχ^2^(20) = 25.03, *p* = 0.200, ΔCFI = 0.001). Adding equal-intercept constraints produced a significant chi-square change (Δχ^2^(20) = 88.92, *p* < 0.001), but ΔCFI = 0.008 and ΔRMSEA = 0.003 remained within commonly used approximate-invariance guidelines (Cheung and Rensvold, 2002). These results support the conclusion that metric invariance was supported and scalar invariance was approximately, rather than exactly, supported. Combined with the consistency in direction when the two institutions were estimated separately, these findings permit comparison of structural relationships in the pooled sample. Invariance across year of study and design specialism is reported in Supplementary Table S2C.

**Table 5:** Measurement invariance tests.

Grouping	Model	χ^2^	df	CFI	RMSEA	Δχ^2^ (Δdf)	*p*	ΔCFI
Institution (2 groups)	Configural invariance	793.43	530	0.965	0.035	—	—	—
	Metric invariance (equal loadings)	819.33	550	0.964	0.034	25.03 (20)	0.200	0.001
	Scalar invariance (equal intercepts)	900.19	570	0.956	0.037	88.92 (20)	< 0.001	0.008

Δχ^2^ and ΔCFI are relative to the preceding model (scaled difference test).

### 3.5 Structural model and tests of H1–H2b and RQ1: parallel dual-outcome model (M1)

The parallel dual-outcome structural equation model (M1) fitted the data well: χ^2^(183) = 383.98, *p* < 0.001, CFI = 0.968, TLI = 0.963, RMSEA = 0.036, 90% CI [0.031, 0.041], SRMR = 0.035. Because its structural part is just-identified, this fit equals that of the corresponding measurement model. The model explained 29.1% of the variance in LE, 51.6% in CSE, and 35.3% in CPI. Table 6 and Figure 2 present the structural paths. The corresponding class-clustered composite-score sensitivity results appear under Robustness checks and in Supplementary Table S4A.

**Table 6:** Structural path estimates for Model M1: parallel comparison of CSE and CPI.

Path	*B*	*β*	Bootstrap SE (β)	*z*	*p*	95% BC CI (β)
PL → LE	0.438	0.539	0.031	17.32	<0.001	[0.473, 0.595]
LE → CSE	0.839	0.618	0.037	16.69	<0.001	[0.541, 0.690]
LE → CPI	0.577	0.474	0.046	10.28	<0.001	[0.383, 0.570]
PL → CSE (direct)	0.179	0.163	0.045	3.62	<0.001	[0.073, 0.248]
PL → CPI (direct)	0.183	0.185	0.053	3.52	<0.001	[0.082, 0.286]
Path difference: LE → CSE vs. LE → CPI	ΔB = 0.262	Δβ = 0.144	0.048	2.97	0.003	[0.044, 0.235]

B, unstandardized; β, standardized. SEs and bias-corrected 95% CIs are from 2,000 class-level bootstrap draws.

**Figure 2:**
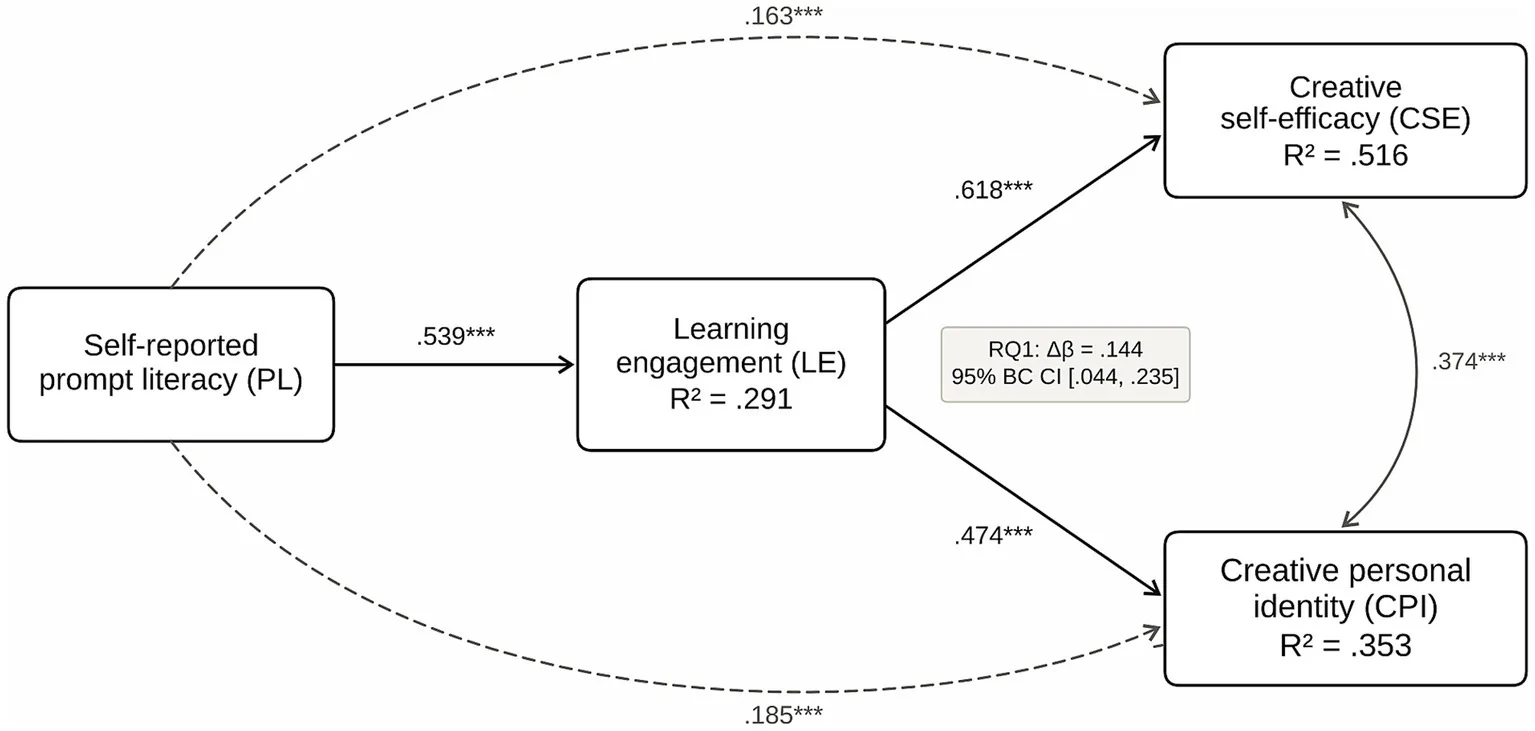
Standardized results of the main model (M1). Dashed curves are direct paths; the double-headed arrow is the CSE–CPI residual correlation. ***p < 0.001 (2,000 class-level bootstrap resamples).

All inferences below are based on 2,000 class-level bootstrap resamples. Prompt literacy was positively associated with learning engagement (*β* = 0.539, SE = 0.031, 95% CI [0.473, 0.595], *p* < 0.001), supporting H1. Learning engagement was positively associated with both CSE (*β* = 0.618, SE = 0.037, 95% CI [0.541, 0.690], *p* < 0.001) and CPI (*β* = 0.474, SE = 0.046, 95% CI [0.383, 0.570], *p* < 0.001), supporting H2a and H2b. The two direct specification paths were also positive and smaller in magnitude (PL → CSE: *β* = 0.163, SE = 0.045, 95% CI [0.073, 0.248], *p* < 0.001; PL → CPI: *β* = 0.185, SE = 0.053, 95% CI [0.082, 0.286], *p* < 0.001). This pattern describes the coexistence of direct and indirect associations.

For RQ1, the standardized path difference was Δ*β* = 0.144 (bootstrap SE = 0.048, 95% BC CI [0.044, 0.235]; *z* = 2.97, *p* = 0.003), and the unstandardized difference was ΔB = 0.262 (SE = 0.075, 95% BC CI [0.110, 0.404]; z = 3.50, *p* < 0.001). A Wald test of the equality restriction gave the same conclusion [χ^2^(1) = 12.49, *p* < 0.001] but is reported only descriptively because it relies on the cluster-robust information matrix. Engagement was thus more strongly associated with CSE than with CPI. The difference is one of degree: engagement was also clearly associated with CPI.

### 3.6 Indirect associations through engagement and differences between outcomes

Indirect associations through LE were tested using 2,000 class-level bootstrap resamples (Table 7). PL was indirectly associated with CSE through LE (standardized indirect association = 0.333, SE = 0.033, 95% BC CI [0.270, 0.399]), supporting H3a, and with CPI through LE (standardized indirect association = 0.255, SE = 0.032, 95% BC CI [0.197, 0.321]), supporting H3b. As part of RQ1, the difference between the two indirect associations was 0.078 (SE = 0.027, 95% BC CI [0.023, 0.130]; *z* = 2.90, *p* = 0.004); because both share the PL → LE path, this difference mirrors the path difference.

**Table 7:** Class-level bootstrap tests of indirect associations (2,000 resamples).

Effect	Standardized estimate	Bootstrap SE	95% BC CI	Conclusion
PL → LE → CSE (H3a)	0.333	0.033	[0.270, 0.399]	Supports H3a
PL → LE → CPI (H3b)	0.255	0.032	[0.197, 0.321]	Supports H3b
Indirect-association difference (RQ1)	0.078	0.027	[0.023, 0.130]	Stronger for CSE
PL → CSE direct	0.163	0.045	[0.073, 0.248]	Significant
PL → CPI direct	0.185	0.053	[0.082, 0.286]	Significant
PL → CSE total association	0.496	0.034	[0.428, 0.560]	Significant
PL → CPI total association	0.440	0.044	[0.347, 0.518]	Significant

Standardized estimates; SEs and CIs as in Table 6.

### 3.7 Sensitivity analysis: the efficacy-gateway restriction (M2)

M2 restricted the engagement–identity association to run entirely through CSE (PL → LE → CSE → CPI, retaining the direct PL paths), an ordering suggested by the statistical AI literacy–CSE–CPI pathway reported by Li H. et al. (2026). M2 fitted acceptably [χ^2^(184) = 394.71, CFI = 0.966, TLI = 0.962, RMSEA = 0.037, SRMR = 0.036; Table 8]. Because M2 is nested in M1′, the single restriction LE → CPI = 0 could be tested directly, and it was rejected [scaled Δχ^2^(1) = 9.89, *p* = 0.002, computed from the Mplus log-likelihoods; 9.86 in lavaan]. In M1′, engagement remained associated with CPI when CSE was taken into account (*β* = 0.21, 95% BC CI [0.09, 0.32], class-level bootstrap in R), alongside the CSE → CPI path (*β* = 0.43). The data therefore do not support complete transmission of the engagement–identity association through efficacy. They do not show that CSE precedes CPI: M1, M1′, and a model with CPI → CSE fit identically, so the ordering of the two beliefs cannot be identified from these data.

**Table 8:** Comparison of the parallel model (M1) and the restricted efficacy-gateway model (M2).

Model	χ^2^	df	CFI	TLI	RMSEA	SRMR	AIC	BIC
M1 parallel dual-outcome	383.98	183	0.968	0.963	0.036	0.035	50512.31	50838.01
M2 efficacy gateway (LE → CSE → CPI)	394.71	184	0.966	0.962	0.037	0.036	50522.12	50843.10

M2 drops LE → CPI and replaces the CSE–CPI residual correlation with CSE → CPI. Tested against the equivalent model M1′, the restriction LE → CPI = 0 gives scaled Δχ^2^(1) = 9.89, *p* = 0.002.

### 3.8 Differential moderation by dimensions of AI dependence

Moderation analyses used standardized composite scores. The simultaneous four-dimension model was the sole primary model: the tests of H4a–H4c, the pairwise comparisons, and the simple slopes came from that model. Total-score and separate single-dimension models are exploratory. All models treated the 40 intact classes as primary sampling units and were estimated with 2,000 class-level bootstrap draws; bias-corrected 95% confidence intervals are reported for every interaction coefficient and contrast. The largest VIF in the simultaneous model was 1.75. The four interactions jointly explained Δ*R*^2^ = 0.051 (*f*^2^ = 0.068). Cognitive dependence uniquely explained Δ*R*^2^ = 0.015 (*f*^2^ = 0.020), loss of control Δ*R*^2^ = 0.006 (*f*^2^ = 0.008), and functional and emotional dependence 0.001 and less than 0.001, respectively. The local effect sizes are small; the evidence concerns the pattern of differences rather than large effects.

#### 3.8.1 Primary analysis: simultaneous four-dimension model

When all four main effects and PL-by-dimension interactions entered together, the pattern was differentiated but small (Table 9). Cognitive dependence had a negative interaction (*B* = −0.155, bootstrap SE = 0.043, *z* = −3.58, *p* < 0.001, 95% BC CI [−0.236, −0.069]), supporting H4a. Loss of control also had a negative interaction (*B* = −0.093, bootstrap SE = 0.040, *z* = −2.31, *p* = 0.021, 95% BC CI [−0.174, −0.011]), supporting H4b in the primary analysis. The unique interactions for functional dependence (*B* = −0.034, *p* = 0.396) and emotional dependence (*B* = 0.003, *p* = 0.941) were not significant. The combined contrast for H4c was negative (−0.218, bootstrap SE = 0.086, *z* = −2.52, *p* = 0.012, 95% BC CI [−0.383, −0.047]). With CR2 standard errors and Satterthwaite degrees of freedom, the conclusions were unchanged: cognitive dependence, *t*(30.1) = −3.64, *p* = 0.001; loss of control, *t*(30.8) = −2.34, *p* = 0.026; H4c contrast, t(32.3) = −2.57, *p* = 0.015. Both hypothesized interactions remained significant after Holm correction for the two directional hypotheses, but the loss-of-control interaction did not survive Holm correction across all four interaction terms (adjusted *p* = 0.063 based on the bootstrap *p* values and 0.078 based on the CR2 *p* values). Exploratory pairwise contrasts showed that cognitive dependence differed from emotional dependence (Δ = −0.158, 95% BC CI [−0.284, −0.036]) and from functional dependence (Δ = −0.121, [−0.236, −0.005]; borderline with CR2 inference, *p* = 0.051), whereas every contrast involving loss of control included zero. H4c was therefore partially supported: the combined contrast was driven mainly by cognitive dependence, with only directionally consistent evidence from loss of control. Figure 3 plots the simple slopes at ±1 SD of cognitive dependence and loss of control.

**Table 9:** Differential moderation by dimensions of AI dependence: simultaneous model.

Model and term	*B*	Bootstrap SE	*z*	*p*	95% BC CI
Simultaneous four-dimension model *R*^2^ = 0.252					
PL	0.452	0.030	15.21	< 0.001	
Emotional dependence	−0.016	0.039	−0.41	0.685	
Functional dependence	0.002	0.036	0.05	0.960	
Cognitive dependence	−0.010	0.037	−0.28	0.784	
Loss of control	0.039	0.037	1.06	0.288	
PL × Emotional dependence	0.003	0.045	0.07	0.941	[−0.087, 0.089]
PL × Functional dependence	−0.034	0.040	−0.85	0.396	[−0.112, 0.044]
PL × Cognitive dependence (H4a)	−0.155	0.043	−3.58	<0.001	[−0.236, −0.069]
PL × Loss of control (H4b)	−0.093	0.040	−2.31	0.021	[−0.174, −0.011]
Combined contrast (H4c): (cognitive + loss of control) − (functional + emotional)	−0.218	0.086	−2.52	0.012	[−0.383, −0.047]
Simple slopes (from the same simultaneous model; other dimensions held at their means)					
PL → LE | Cognitive dependence −1 SD	0.607	0.049	12.32	<0.001	[0.506, 0.702]
PL → LE | Cognitive dependence +1 SD	0.297	0.056	5.35	<0.001	[0.192, 0.408]
PL → LE | Loss of control −1 SD	0.546	0.050	10.85	<0.001	[0.450, 0.647]
PL → LE | Loss of control +1 SD	0.359	0.050	7.16	<0.001	[0.266, 0.465]
Increment from interaction block: Δ*R*^2^ = 0.051 (*f*^2^ = 0.068)					
Unique increment of PL × cognitive dependence	Δ*R*^2^ = 0.015	*f*^2^ = 0.020			
Unique increment of PL × loss of control	Δ*R*^2^ = 0.006	*f*^2^ = 0.008			
Unique increment of PL × functional dependence	Δ*R*^2^ = 0.001	*f*^2^ = 0.001			
Unique increment of PL × emotional dependence	Δ*R*^2^ < 0.001	*f*^2^ < 0.001			

Estimates from the simultaneous four-dimension model (standardized variables). SEs, z, and bias-corrected 95% CIs, including those for the simple slopes, are from the class-level bootstrap.

**Figure 3:**
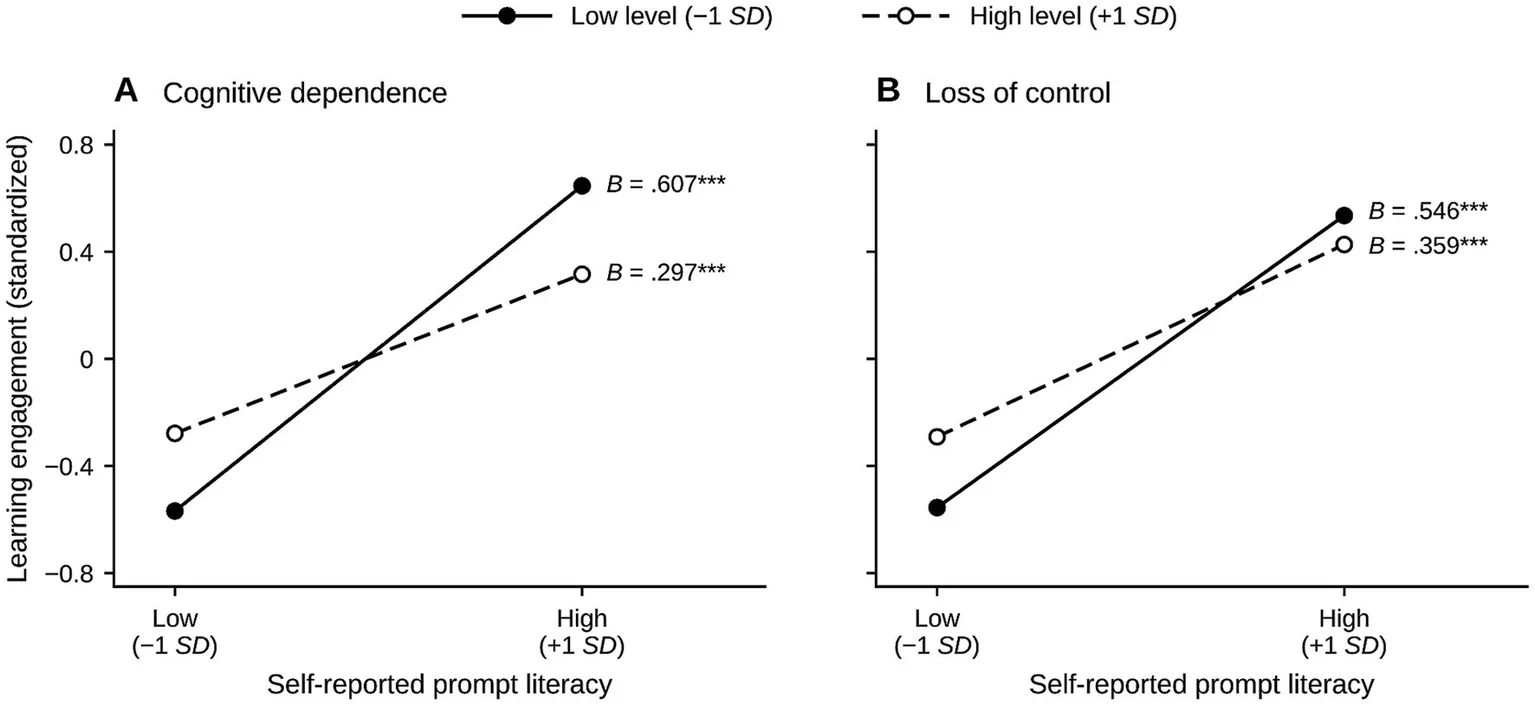
Simple slopes of self-reported prompt literacy on learning engagement at low (−1 SD) and high (+1 SD) **(A)** cognitive dependence and **(B)** loss of control, from the simultaneous four-dimension model. Confidence intervals are given in Table 9. ***p < 0.001.

**Exploratory and sensitivity analyses:** total-score, single-dimension, and covariate-adjusted models. The interaction with the total AID score was negative (*B* = −0.202, bootstrap SE = 0.035, *p* < 0.001, 95% BC CI [−0.268, −0.136]), but the total score could not distinguish dimensions. All four interactions were negative and significant when tested separately (Supplementary Table S5A); only cognitive dependence and loss of control retained unique interactions in the simultaneous model. Because the dimensions correlated at *r* = 0.43–0.50, this change was consistent with shared variance, although the loss of significance alone could not show that the separate effects arose entirely from shared variance. Figure 4 displays the change in estimates without labelling any dimension risk-free. In a model adjusted for institution, sex, year of study, design specialism, and weekly GenAI use (all dummy-coded; CR2 inference), the interactions for cognitive dependence (*B* = −0.157, *p* < 0.001) and loss of control (*B* = −0.097, *p* = 0.022) were essentially unchanged. Adding the interaction between PL and weekly use frequency did not alter them (cognitive dependence *B* = −0.158, *p* < 0.001; loss of control *B* = −0.097, *p* = 0.024), and that interaction was negligible (*B* = −0.012, *p* = 0.75). Weekly use was also unrelated to engagement (*r* = −0.02) and to loss of control (*r* = 0.04). The loss-of-control interaction was thus not explained by how often students used GenAI.

**Figure 4:**
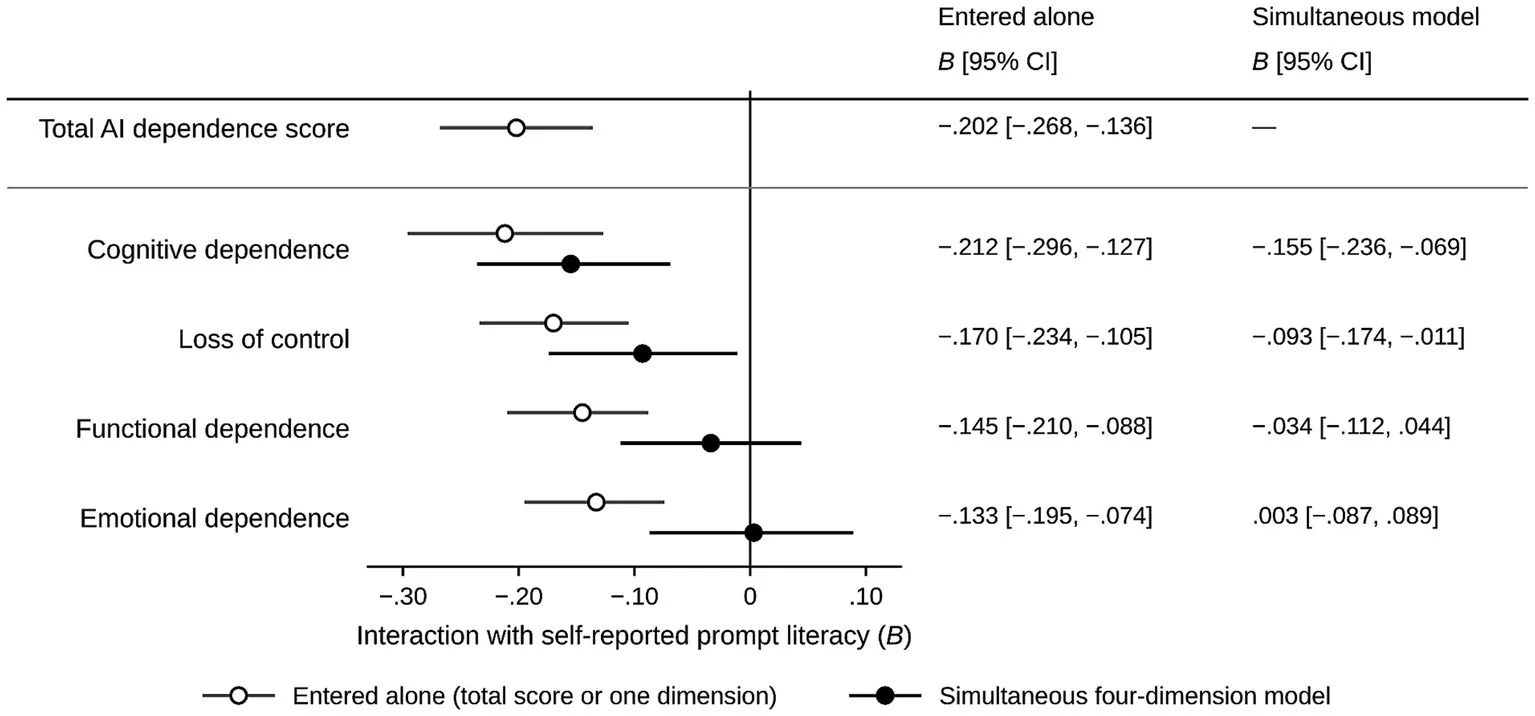
Interaction coefficients (B) for self-reported prompt literacy with the total AI-dependence score and each dimension. Open circles: entered alone; filled circles: simultaneous four-dimension model. Lines show bias-corrected 95% CIs (2,000 class-level bootstrap draws).

#### 3.8.2 Simple slopes

At +1 and −1 SD of cognitive dependence, the simple slopes of PL on LE were 0.297 and 0.607; at +1 and −1 SD of loss of control, they were 0.359 and 0.546 (all *p* < 0.001; Table 9). The association therefore remained positive at higher levels of both dimensions: higher dependence was associated with a weaker, not a reversed, association. These values describe conditional associations, not causal effects. Conditional indirect associations and indices of moderated mediation are exploratory and are reported in Supplementary Table S5B.

### 3.9 Robustness checks

#### 3.9.1 Re-estimation without performance engagement

Because performance engagement contained only two items and had lower reliability (α = 0.666), M1 was re-estimated without this dimension. Model fit and path patterns were unchanged (CFI = 0.967, RMSEA = 0.038; PL → LE = 0.535, LE → CSE = 0.619, LE → CPI = 0.480), and the path difference remained in the direction found for RQ1.

#### 3.9.2 Covariate adjustment and institution as a fixed control

In a composite-score path model adjusted for institution, sex, year of study, design specialism, and weekly GenAI use (all dummy-coded), all primary paths remained significant with CR2 inference (PL → LE = 0.440; LE → CSE = 0.494; PL → CSE = 0.201; LE → CPI = 0.375; PL → CPI = 0.204; all *p* < 0.001), and LE → CSE exceeded LE → CPI (difference = 0.119, 95% CI [0.041, 0.197]). In the latent M1 with all four latent variables regressed on the same covariates, the fully standardized estimates were PL → LE = 0.535 (95% BC CI [0.463, 0.591]), LE → CSE = 0.618 ([0.543, 0.694]), and LE → CPI = 0.475 ([0.378, 0.562]); Δβ = 0.143 ([0.037, 0.243]), and the difference between the indirect associations was 0.076 ([0.017, 0.132]) (2,000 class-level bootstrap resamples). With only two institutions, random effects could not be estimated, so institution was treated as a fixed control.

#### 3.9.3 Cross-institutional replication

M1 fitted acceptably in each institution (Institution A: *n* = 573, CFI = 0.970, RMSEA = 0.036; Institution B: *n* = 256, CFI = 0.960, RMSEA = 0.040). The three core paths were positive in both institutions (A: PL → LE = 0.520, LE → CSE = 0.607, LE → CPI = 0.454; B: PL → LE = 0.564, LE → CSE = 0.637, LE → CPI = 0.524), and LE → CSE exceeded LE → CPI in each. The direct paths were positive in both institutions (A: PL → CSE = 0.160, PL → CPI = 0.185; B: PL → CSE = 0.181, PL → CPI = 0.201). Because the two subsamples contain only 28 and 12 classes, cluster-robust standard errors are not trustworthy at that level, so these institution-specific estimates are reported as descriptive replication rather than as significance tests; on that basis the model was reproduced in both institutions.

#### 3.9.4 Sensitivity of clustered inference and robustness summary

Comparing the 2,000-draw class-level bootstrap with a 2,000-draw individual-level bootstrap across parameters yielded SE ratios of 0.94–1.16 across the 12 estimated quantities (PL → LE 0.94, LE → CSE 0.95, LE → CPI 1.12, Δβ 1.04, difference between the indirect associations 1.08, total association with CPI 1.16). Consistent with ICC ≤ 0.041, ignoring clustering did not systematically underestimate uncertainty here. Nevertheless, all reported intervals used the class-level bootstrap, and conclusions for H1–H3b and RQ1 were identical under both resampling schemes. A standardized composite-score model with 2,000 class-level bootstrap draws produced PL → LE = 0.447 (95% CI [0.387, 0.494]), LE → CSE = 0.495 ([0.445, 0.542]), and LE → CPI = 0.374 ([0.311, 0.441]); the indirect associations were 0.221 and 0.167, with a difference of 0.054 (95% CI [0.020, 0.085]). These coefficients were smaller because composite models did not correct for measurement error, but their directions and significance agreed with M1 (Supplementary Table S4A). With CR2 inference, the composite-score path difference was 0.121 (95% CI [0.045, 0.197], *p* = 0.003). Leave-one-class-out analyses indicated that no single class determined the results: across the 40 re-estimations of M1, PL → LE ranged from 0.529 to 0.557, LE → CSE from 0.609 to 0.633, LE → CPI from 0.456 to 0.491, and Δβ from 0.129 to 0.170; in the moderation model, the cognitive-dependence interaction ranged from −0.176 to −0.144 (all CR2 *p* < 0.005), and the loss-of-control interaction ranged from −0.111 to −0.079 and remained significant in every re-estimation, although its CR2 *p-*value exceeded 0.04 in eight of them. Together with the moderation analyses reported above, these checks indicate that the main patterns were stable across specifications, with the loss-of-control interaction the least robust. Table 10 summarizes the tests of the hypotheses and RQ1.

**Table 10:** Summary of hypothesis tests and the research question.

Hypothesis or question	Path/test	Estimate (β or B)	95% CI / p	Conclusion
H1	PL → LE	*β* = 0.539	[0.473, 0.595]	Supported
H2a	LE → CSE	*β* = 0.618	[0.541, 0.690]	Supported
H2b	LE → CPI	*β* = 0.474	[0.383, 0.570]	Supported
RQ1	LE → CSE stronger than LE → CPI	Δ*β* = 0.144	[0.044, 0.235], *p* = 0.003	Yes; difference in degree
H3a	PL → LE → CSE	*β* = 0.333	[0.270, 0.399]	Supported
H3b	PL → LE → CPI	*β* = 0.255	[0.197, 0.321]	Supported
RQ1 (indirect)	Indirect-association difference (CSE > CPI)	Δ = 0.078	[0.023, 0.130], *p* = 0.004	Yes; mirrors the path difference
H4a	PL × Cognitive dependence → LE	*B* = −0.155	Bootstrap SE = 0.043, *p* < 0.001; 95% BC CI [−0.236, −0.069]	Supported (f^2^ = 0.020, small effect)
H4b	PL × Loss of control → LE	*B* = −0.093	Bootstrap SE = 0.040, *p* = 0.021; 95% BC CI [−0.174, −0.011]	Supported, weaker evidence (f^2^ = 0.008; not significant after Holm correction across four interactions)
H4c	(cognitive + loss of control) more negative than (functional + emotional)	Contrast = −0.218	SE = 0.086, *p* = 0.012; 95% BC CI [−0.383, −0.047]	Partially supported; driven mainly by cognitive dependence; small effect requiring replication
(specification path)	PL → CSE direct	*β* = 0.163	[0.073, 0.248]	Significant
(specification path)	PL → CPI direct	*β* = 0.185	[0.082, 0.286]	Significant
(structural sensitivity)	Restriction LE → CPI = 0 (M2 vs. M1′)	Scaled Δχ^2^(1) = 9.89	*p* = 0.002	Complete transmission through efficacy not supported; the ordering of CSE and CPI is not identifiable

## 4 Discussion

This study asked how undergraduate art and design students’ self-reported capability to work with GenAI relates to their engagement in design learning, how engagement relates to two components of the creative self, and under which forms of AI dependence the capability–engagement association is weaker. Three findings stand out. Self-reported prompt literacy was positively associated with engagement; engagement was more closely associated with creative self-efficacy than with creative personal identity; and the prompt literacy–engagement association was weaker among students reporting higher cognitive dependence, with weaker evidence for loss of control. In keeping with the cross-sectional, self-reported design, we discuss these findings as associations and possible boundary conditions.

### 4.1 Unequal associations of engagement with efficacy and identity

Prompt literacy was positively associated with engagement, and engagement with both creative self-beliefs. This pattern is consistent with social cognitive theory’s account of personal capability, behavior, and self-beliefs as mutually related (Bandura, 1986, 1997) and with survey evidence from other student populations linking information literacy (Zhao, 2024) and GenAI use (Guo et al., 2025) to engagement. It extends research on AI literacy and the creative self (Li H. et al., 2026; Peng et al., 2026) by placing learning engagement between GenAI-related capability and creative self-beliefs. The design leaves two related uncertainties. Cross-sectional indirect associations cannot establish temporal order or exclude reverse relationships and unmeasured confounding (Maxwell and Cole, 2007; Rohrer et al., 2022); engagement may, for example, also build students’ prompting capability. In addition, students who evaluate themselves favorably in one domain may do so in others, so part of the association between self-reported prompt literacy and engagement may reflect general self-evaluation, which self-report data cannot separate from the constructs of interest.

The comparison addressed by RQ1 is the study’s most specific result. Engagement was more strongly associated with CSE than with CPI. The difference was in the same direction in both institutions and held after covariate adjustment, in composite-score models, and in every leave-one-class-out estimate. Social cognitive theory offers one interpretation: engagement supplies mastery experiences that bear directly on beliefs about what one can do (Bandura, 1997), whereas creative identity also depends on how much students value creativity and on longer-term self-definition (Eccles, 2009). The finding qualifies studies that combined the two beliefs into a single composite (Peng et al., 2026), because with engagement in view the two components were not interchangeable. It also extends work that placed identity downstream of efficacy (Li H. et al., 2026): engagement retained a unique association with identity after efficacy was taken into account, although these data cannot establish which belief comes first. Two cautions apply. The difference is one of degree, since engagement was also clearly associated with CPI. And because LE and CSE were the least distinct pair in the measurement model, part of the stronger engagement–efficacy association may reflect overlap between the two measures; the composite and covariate-adjusted analyses use the same self-reports and cannot remove shared content. Whether ambiguity about authorship in GenAI-supported work, a concern raised in work on GenAI in design and creative contexts (Bartlett and Camba, 2024; Heigl, 2026), contributes to the weaker identity association could be examined by measuring students’ attributions of authorship directly.

### 4.2 Dependence dimensions associated with a weaker literacy–engagement link

The dimensional analysis was most informative for cognitive dependence. The clearest evidence came from cognitive dependence: its interaction held under the class-level bootstrap, CR2 tests, covariate adjustment, and the omission of any single class, and in exploratory contrasts it differed from the interaction for emotional dependence and, less clearly, from that for functional dependence. The loss-of-control interaction was smaller; it was significant in the primary and CR2 tests and after covariate adjustment, but not after correction across all four interaction terms, and it did not differ reliably from the other dimensions. H4c was therefore only partially supported. The total-score interaction was negative, and every dimension interacted significantly with PL when tested alone, but only cognitive dependence and loss of control retained unique interactions when the dimensions were modeled together. Distinguishing the dimensions thus revealed differences that the total score masked, with the clearest unique moderation coming from cognitive dependence.

This pattern fits the social cognitive distinction between personal and proxy agency. Cognitive dependence describes letting AI think and judge in one’s place, the form of reliance most directly opposed to personal agency (Bandura, 2000, 2001), whereas functional delegation need not displace students’ own thinking about a task, and emotional reliance concerns comfort rather than the substitution of thought. The pattern is also consistent with survey evidence that cognitive dependence is more strongly associated with cognitive inertia than tool dependence (Yang et al., 2025) and with concerns about cognitive erosion and dependence among university students (Jose et al., 2025; Zhang and Xu, 2025; Wang et al., 2026). The simple slopes indicate a weaker rather than a reversed association: even at high cognitive dependence, PL remained positively associated with engagement. The local effect sizes were small, and the absence of unique moderation for functional and emotional dependence does not show that these forms of reliance are harmless; their intervals remained compatible with small negative interactions (lower limits −0.112 and −0.087). For research on motivation and engagement, these results identify cognitive dependence as a possible boundary condition for the link between GenAI-related capability and engagement, one that requires replication.

### 4.3 Stability across two institutions: boundaries of the evidence

Sampling 40 classes across two institutions partly reduced the limitations of a single-site study. Metric invariance was fully supported and scalar invariance was approximately supported. Core and direct paths had similar directions and magnitudes in each institutional subsample, and the stronger efficacy than identity path was reproduced in both. The main structural findings were consistent across the class-level bootstrap, CR2 tests of the composite-score model, and leave-one-class-out analyses. The cognitive-dependence interaction also held across the robustness checks. The boundary of this evidence is important: with one institution per sector, institution and sector cannot be separated, so the stability reported here is stability within these two institutions rather than across sectors. Institution could only be a fixed control because two sites were insufficient for a random institutional level. Testing sector-level moderation will require multiple institutions in each sector and multilevel modeling at the institutional level.

### 4.4 Theoretical implications

The findings have two theoretical implications. First, they refine accounts that treat creative self-beliefs as a single composite. In GenAI-supported design learning, engagement was more closely associated with the motivational belief that one can be creative than with the importance of creativity to one’s identity, although it was associated with both. For social cognitive theory, this is consistent with the view that engagement, as a source of mastery experiences, bears most directly on efficacy beliefs (Bandura, 1997); for research on the creative self, it indicates that the two components should be examined separately when their relations with learning processes are of interest (Karwowski, 2012; Karwowski et al., 2018). Because the closest prior studies either combined the two beliefs (Peng et al., 2026) or modeled identity as an outcome of efficacy (Li H. et al., 2026), this comparison is new rather than a replication. However, the low AVE values and the overlap between LE and CSE limit how confidently this difference can be interpreted. Second, distinguishing the dimensions of AI dependence revealed differences that the total score masked: the total-score interaction was negative, but the clearest unique moderation came from cognitive dependence, and the evidence for loss of control was weaker. This links research on AI dependence to the social cognitive distinction between personal and proxy agency (Bandura, 2001) and suggests that studies of dependence and learning may benefit from modeling its dimensions jointly rather than relying on a total score. Methodologically, the study illustrates how class-nested survey data can be analyzed with resampling and small-sample corrections that match inference to the number of clusters.

### 4.5 Practical implications

The findings suggest several pedagogical possibilities. Because none was tested here and the interaction effects were small, we present them as possibilities for evaluation rather than as recommendations. Prompt training could ask students not only to write better prompts but also to compare alternatives, interpret outputs, and revise iteratively, so that the capability is exercised rather than delegated. Because cognitive dependence marked the clearest weakening of the association between perceived prompting capability and engagement, instructors could try activities that keep students’ own thinking in the process, such as recording an initial idea before consulting AI, justifying why an AI-generated proposal was accepted or rejected, and keeping process logs of their own decisions. And because engagement was less closely associated with identity than with efficacy, authorship statements and process portfolios in which students describe what AI contributed and what they decided could be evaluated as ways of supporting creative identity. Whether such practices change engagement, prompting performance, or the quality of creative work is a question for intervention studies.

### 4.6 Limitations and future directions

Several features of the design delimit the conclusions. First, the data are cross-sectional, so they cannot show temporal order, change, or causal effects; the indirect associations describe how the measured variables relate within one specification, not mediating processes (Maxwell and Cole, 2007; Rohrer et al., 2022). Second, all constructs were self-reported by the same students. The diagnostics did not indicate a dominant method factor, but shared method variance and general self-evaluation may still inflate the associations among the self-reported constructs, and the moderation results cannot be assumed to be free of them. Third, prompt literacy was measured with adapted self-report items (Liu X. et al., 2025; Tour and Zadorozhnyy, 2025; Yaşar et al., 2025), so the findings concern perceived prompting capability rather than demonstrated competence, and the measure’s distinctiveness from AI self-efficacy and general digital confidence could not be tested. Fourth, AVE fell below 0.50 for PL, AID, CSE, and CPI, and LE and CSE did not meet the Fornell–Larcker criterion. For AID, the moderate correlations among its four dimensions leave substantial dimension-specific variance unexplained by a single factor, the same property that makes the dimensional tests informative; for the three short item-level scales, heterogeneous item content and translation are plausible contributors, as is the change of response format for CSE and CPI, and PL spans three interaction steps across six items. The SSCS was contextualized to design learning through the instructions only, so dedicated contextual validation is still needed, and the comparison of the two engagement paths should be read alongside the borderline LE–CSE result. Fifth, both institutions were in mainland China, institution and sector were confounded, and the samples differed in year of study and specialism. The findings therefore pertain to undergraduate art and design students in blended, GenAI-supported courses at these two institutions and should not be generalized to other disciplines, educational systems, cultural contexts, or forms of AI-supported learning without replication (Simons et al., 2017). Sixth, 40 classes support class-level resampling and small-sample corrections but limit the precision of interaction estimates and preclude stable estimation of class-level random slopes. Seventh, the interaction effects were small, and the evidence for loss of control was weaker than that for cognitive dependence. Future studies could combine objective prompting tasks, platform logs, behavioral indicators of engagement, and blind assessments of creative products with longitudinal or experimental designs in larger multi-institutional samples, preferably with preregistered hypotheses for the dependence dimensions.

## 5 Conclusion

This study asked how undergraduate art and design students’ self-reported capability to work with GenAI relates to their learning engagement and creative self-beliefs, and under which forms of AI dependence that link is weaker. Self-reported prompt literacy was positively associated with engagement, and engagement was more closely associated with creative self-efficacy than with creative personal identity, while remaining associated with both. Among the four dimensions of AI dependence, cognitive dependence provided the clearest evidence of a weaker prompt literacy–engagement association; evidence for loss of control was weaker, and all interaction effects were small. For research on GenAI, motivation, and engagement, the findings suggest that students’ perceived capability to work with GenAI goes together with engagement less closely among students who report letting AI think in their place, and that engagement aligns more closely with what students believe they can do than with who they believe they are. Given the cross-sectional, self-report design and the measurement limitations, these associations require replication with longitudinal and behavioral data.

## Data Availability

The original contributions presented in the study are included in the article/Supplementary material. The raw data supporting the conclusions of this article are available from the corresponding author upon request.
