# Supplementary material for: Learning engagement and creative self-beliefs in generative AI-supported design education: self-reported prompt literacy and dimensions of AI dependence
Source: Front Psychol. 2026 Oct 28;17:1957097. doi: 10.3389/fpsyg.2026.1957097 (PMC13752757; doi:10.3389/fpsyg.2026.1957097)
Supplement: Supplementary file 1 [file Supplementary_file_1.docx]

Supplementary Material

# 1 Supplementary Tables S1–S6

**Table S1. Pilot study (N = 104): item analysis and joint exploratory factor analysis**

**Panel A. Item analysis of the five instruments**

| **Construct** | **Items** | **Critical ratio CR** | **CITC** | **α** | **Note** |
| --- | --- | --- | --- | --- | --- |
| Prompt literacy, PL | 6 | 9.32–13.89 | .61–.78 | .878 |  |
| Learning engagement, LE | 19 | 5.89–10.93 | .46–.72 | .934 |  |
| AI dependence, AID | 22 | 6.08–9.53 | .44–.66 | .935 |  |
| Creative self-efficacy, CSE | 6 | 7.48–10.48 | .55–.71 | .848 |  |
| Creative personal identity, CPI | 5 | 6.32–11.83 | .52–.74 | .812 |  |

**Panel B. Joint exploratory factor analysis of the prompt-literacy and creative-self items**

| **Item** | **Pattern F1 (PL)** | **Pattern F2 (CSE)** | **Pattern F3 (CPI)** | **Structure F1** | **Structure F2** | **Structure F3** | **Communality h²** |
| --- | --- | --- | --- | --- | --- | --- | --- |
| PL1 | **.752** | .094 | −.102 | .764 | .259 | .039 | .595 |
| PL2 | **.758** | −.136 | .219 | .750 | .162 | .266 | .605 |
| PL3 | **.671** | −.001 | .010 | .672 | .188 | .101 | .451 |
| PL4 | **.679** | −.063 | −.011 | .660 | .120 | .056 | .440 |
| PL5 | **.756** | .038 | −.029 | .762 | .234 | .089 | .582 |
| PL6 | **.829** | .082 | −.077 | .841 | .279 | .070 | .715 |
| CSE1 | .055 | **.614** | .097 | .238 | .669 | .356 | .459 |
| CSE2 | .020 | **.662** | −.030 | .198 | .655 | .243 | .430 |
| CSE3 | .092 | **.596** | −.052 | .249 | .600 | .205 | .370 |
| CSE4 | −.026 | **.679** | .100 | .175 | .713 | .374 | .517 |
| CSE5 | −.140 | **.816** | .002 | .085 | .779 | .317 | .625 |
| CSE6 | −.010 | **.793** | −.086 | .197 | .755 | .237 | .576 |
| CPI1 | .006 | −.071 | **.885** | .107 | .293 | .857 | .739 |
| CPI2 | .010 | −.010 | **.742** | .109 | .296 | .739 | .547 |
| CPI3 | −.004 | .225 | **.478** | .124 | .420 | .570 | .366 |
| CPI4 | .021 | −.020 | **.608** | .098 | .235 | .603 | .364 |
| CPI5 | −.043 | −.021 | **.672** | .043 | .243 | .658 | .436 |
| Cronbach’s α | PL = .878 | CSE = .848 | CPI = .812 |  |  |  |  |

**Note.** N = 104 in an independent pilot sample that did not overlap with the main sample (N = 829). Panel A: CR = critical ratio from independent-samples t tests comparing the upper and lower 27% groups; all item CRs had p < .001. CITC = corrected item–total correlation; α = Cronbach’s α. Exploratory factor analysis jointly examined the 11 newly translated SSCS items and the six adapted prompt-literacy items; the learning-engagement and AI-dependence instruments proceeded directly to confirmatory factor analysis in the main sample because AIDep-22 was developed with Chinese undergraduates and the OSE has an established four-dimension structure. Panel B: Principal-axis factoring with Promax rotation (κ = 4) and Kaiser normalization; three factors were extracted. KMO = .829; Bartlett χ²(136) = 761.2, p < .001. Eigenvalues were 5.02, 3.13, 2.04, and 0.86; parallel analysis (95th percentile, 1,000 replications) retained three factors, with thresholds of 1.93, 1.72, and 1.57 for the first three components. The three factors explained 60.0% of the variance (initial-eigenvalue basis). Factor correlations were .28 (PL–CSE), .14 (PL–CPI), and .41 (CSE–CPI). Bold values are primary loadings; every item loaded primarily on its intended factor, the largest cross-loading was .225 (CPI3 on the CSE factor), and none reached .25. Because the ratio of cases to items was 6.1:1, this pilot analysis is exploratory; the item-level confirmatory factor analysis and HTMT results in the main sample provide the primary evidence for the measurement structure.

**Table S2. Additional measurement-model results: first-order measurement quality, parcel loadings, and invariance across year and specialism**

**Panel A.** First-order dimension reliability and convergent validity

| **1st-order dimension** | **Item range** | **Items** | **M** | **SD** | **α** | **CR** | **AVE** | **Item loading range** |
| --- | --- | --- | --- | --- | --- | --- | --- | --- |
| Skills engagement | LE1–LE6 | 6 | 5.00 | 0.95 | .814 | .814 | .422 | .591–.689 |
| Emotional engagement | LE7–LE11 | 5 | 5.07 | 0.89 | .739 | .739 | .363 | .546–.676 |
| Participation engagement | LE12–LE17 | 6 | 5.10 | 1.01 | .852 | .853 | .491 | .677–.719 |
| Performance engagement | LE18–LE19 | 2 | 5.30 | 1.09 | .666 | .668 | .502 | .685–.731 |
| Emotional dependence | OR1–OR5 | 5 | 3.66 | 0.97 | .803 | .809 | .461 | .561–.759 |
| Functional dependence | OR6–OR11 | 6 | 3.76 | 1.05 | .837 | .839 | .466 | .616–.758 |
| Cognitive dependence | OR12–OR16 | 5 | 3.39 | 1.03 | .815 | .815 | .470 | .628–.772 |
| Loss of control | OR17–OR22 | 6 | 3.59 | 0.99 | .814 | .818 | .431 | .577–.755 |

**Panel B.** Loadings of the dimension-mean parcels on LE and AID

| **Construct** | **Dimension** | **Std. loading** |
| --- | --- | --- |
| Learning engagement, LE | Skills engagement | .747 |
|  | Emotional engagement | .769 |
|  | Participation engagement | .749 |
|  | Performance engagement | .683 |
| AI dependence, AID | Emotional dependence | .708 |
|  | Functional dependence | .684 |
|  | Cognitive dependence | .645 |
|  | Loss of control | .653 |

**Panel C. Measurement invariance across year-of-study and design-specialism groups**

| **Grouping** | **Model** | **χ²** | **df** | **CFI** | **RMSEA** | **Δχ² (Δdf)** | **p** | **ΔCFI** |
| --- | --- | --- | --- | --- | --- | --- | --- | --- |
| Year of study (3 groups) | Configural invariance | 1101.27 | 795 | .960 | .037 | — | — | — |
|  | Metric invariance | 1158.04 | 835 | .958 | .037 | 57.03 (40) | .039 | −.002 |
| Design specialism (4 groups) | Configural invariance | 1603.02 | 1060 | .934 | .051 | — | — | — |
|  | Metric invariance | 1674.05 | 1120 | .932 | .050 | 67.61 (60) | .233 | −.002 |

**Note.** LE1–LE19, learning-engagement items; OR1–OR22, AI-dependence items. Panel C: Satorra–Bentler scaled Δχ²; specialism analyses used the four main groups (n = 79–266).

**Table S3. Zero-order correlations between demographic variables and the focal constructs**

| **Zero-order correlations between demographic variables and focal constructs** | **PL** | **LE** | **AI dependence** | **CSE** | **CPI** |
| --- | --- | --- | --- | --- | --- |
| Sex (female = 1) | .040 | .006 | .000 | .011 | −.053 |
| Year of study | −.106** | −.067 | .014 | −.089** | −.072* |
| Age | −.076* | −.059 | −.015 | −.082* | −.075* |
| Weekly GenAI use | .010 | −.018 | .043 | .016 | .012 |
| Institution (Institution B = 1) | .112** | .074* | −.037 | .058 | .008 |

**Note.** Female = 1; year of study = 2–4; weekly GenAI use = 1–4 (ordinal); Institution B = 1. *p < .05, **p < .01.

**Table S4. Robustness of the structural paths: class-clustered composite-score and covariate-adjusted models**

**Panel A. Class-clustered composite-score sensitivity analysis of primary paths and indirect associations**

| **Parameter** | **Standardized B** | **Class-bootstrap 95% CI** |
| --- | --- | --- |
| PL → LE | .447 | [.387, .494] |
| LE → CSE (adjusted for PL) | .495 | [.445, .542] |
| LE → CPI (adjusted for PL) | .374 | [.311, .441] |
| PL → CSE (adjusted for LE) | .202 | [.142, .261] |
| PL → CPI (adjusted for LE) | .201 | [.126, .272] |
| PL → LE → CSE indirect association | .221 | [.179, .262] |
| PL → LE → CPI indirect association | .167 | [.127, .213] |
| Difference between indirect associations | .054 | [.020, .085] |

**Panel B. Covariate-adjusted models and institution as a fixed control**

| **Model and parameter** | **Standardized B** | **95% CI** |
| --- | --- | --- |
| Composite-score path model adjusted for institution, sex, year of study, design specialism, and weekly GenAI use (CR2) | | |
| PL → LE | .440 | [.383, .497] |
| LE → CSE (adjusted for PL) | .494 | [.437, .551] |
| PL → CSE (adjusted for LE) | .201 | [.134, .268] |
| LE → CPI (adjusted for PL) | .375 | [.296, .454] |
| PL → CPI (adjusted for LE) | .204 | [.126, .282] |
| Path difference (LE → CSE − LE → CPI) | .119 | [.041, .197] |
| Latent M1 **adjusted** for institution and the same covariates (2,000 class-level bootstrap draws) | | |
| PL → LE | .535 | [.463, .591] |
| LE → CSE | .618 | [.543, .694] |
| LE → CPI | .475 | [.378, .562] |
| Path difference (Δβ) | .143 | [.037, .243] |
| Difference between indirect associations | .076 | [.017, .132] |

**Note.** N = 829 students in 40 classes. Panel A: standardized composite scores; bias-corrected 95% CIs from 2,000 class-level bootstrap draws (Mplus 8.3). Composite models do not correct for measurement error, so their coefficients are smaller than the latent estimates. Panel B: covariates were dummy-coded; the composite model used CR2 cluster-robust standard errors with Satterthwaite df (all paths p < .001; path difference p = .004), and the latent M1 used 2,000 class-level bootstrap draws (bias-corrected CIs). Institution was entered as a fixed control because only two institutions were available.

**Table S5. Moderation by AI dependence: robustness checks and conditional effects**

**Panel A. Total-score baseline and separate single-dimension models**

| **Model and term** | **B** | **Cluster boot SE** | **z** | **p** | **95% BC CI** |
| --- | --- | --- | --- | --- | --- |
| Baseline model (total score) R² = .243 |  |  |  |  |  |
| PL | .448 | .029 | 15.42 | < .001 |  |
| Total AID score | .006 | .022 | 0.29 | .772 |  |
| PL × Total AID score | −.202 | .035 | −5.86 | < .001 | [−.268, −.136] |
| Separate models (one dimension at a time) R² = .218–.241 |  |  |  |  |  |
| PL × Emotional dependence | −.133 | .031 | −4.33 | < .001 | [−.195, −.074] |
| PL × Functional dependence | −.145 | .031 | −4.69 | < .001 | [−.210, −.088] |
| PL × Cognitive dependence | −.212 | .044 | −4.77 | < .001 | [−.296, −.127] |
| PL × Loss of control | −.170 | .033 | −5.17 | < .001 | [−.234, −.105] |

**Panel B. Conditional indirect associations and indices of moderated mediation**

| **Moderator** | **Pathway** | **Index of moderated mediation** | **95% BC CI** | **Conditional indirect: −1 SD** | **+1 SD** |
| --- | --- | --- | --- | --- | --- |
| Cognitive dependence | PL → LE → CSE | −.077 | [−.118, −.036] | .301 | .147 |
| Cognitive dependence | PL → LE → CPI | −.058 | [−.092, −.025] | .227 | .111 |
| Loss of control | PL → LE → CSE | −.046 | [−.085, −.005] | .270 | .178 |
| Loss of control | PL → LE → CPI | −.035 | [−.067, −.004] | .204 | .134 |
| Functional dependence | PL → LE → CSE | −.017 | [−.054, .023] | **—** | **—** |
| Functional dependence | PL → LE → CPI | −.013 | [−.043, .017] | **—** | **—** |
| Emotional dependence | PL → LE → CSE | .002 | [−.044, .044] | **—** | **—** |
| Emotional dependence | PL → LE → CPI | .001 | [−.032, .036] | **—** | **—** |

**Note.** N = 829 students in 40 classes; all variables are standardized composite scores. Estimates come from Mplus 8.3 (TYPE = COMPLEX, CLUSTER = class) with 2,000 class-level bootstrap draws; intervals are bias-corrected. Panel A: the total score or one dimension was entered alone (exploratory models). Panel B: conditional effects were derived from the simultaneous four-dimension model, with the other dimensions and their interactions held at zero; indices of moderated mediation follow Hayes (2015). Conditional indirect associations are not reported for functional and emotional dependence because their interactions were not significant in that model.

**Table S6. Full item wording of the five instruments (as administered)**

| **Item** | **Construct / interaction step** | **Chinese wording (as administered)** | **English source wording** |
| --- | --- | --- | --- |
| PL1 | PL – crafting prompts | 我能够根据设计任务的要求，设计出有效的AI提示词。 | I can design effective AI prompts based on task requirements. |
| PL2 | PL – crafting prompts | 我能够通过融入专业（设计）术语和示例来优化AI提示词。 | I can optimize AI prompts by integrating technical terminology and examples. |
| PL3 | PL – reviewing and revising prompts | 我能够根据生成结果和反馈，持续改进AI提示词。 | I can continuously refine AI prompts based on generated results and feedback. |
| PL4 | PL – engaging critically with generated responses | 我能够评估AI生成内容的准确性与可靠性。 | I can assess the accuracy and reliability of AI-generated content. |
| PL5 | PL – engaging critically with generated responses | 我能够评估AI生成内容的一致性与连贯性。 | I can assess the consistency and coherence of AI-generated content. |
| PL6 | PL – engaging critically with generated responses | 我能够评估AI生成内容的逻辑性与完整性。 | I can evaluate the logic and completeness of AI-generated content. |
| LE1 | LE – skills | 坚持有规律地学习设计课程内容。 | Making sure to study on a regular basis. |
| LE2 | LE – skills | 及时跟上课程的阅读与案例资料。 | Staying up on the readings. |
| LE3 | LE – skills | 课后复习笔记，以确保自己理解所学的设计内容。 | Looking over class notes between getting online to make sure I understand the material. |
| LE4 | LE – skills | 学习设计时做事有条理。 | Being organized. |
| LE5 | LE – skills | 认真听讲、仔细阅读学习材料。 | Listening/reading carefully. |
| LE6 | LE – skills | 针对阅读材料、课件或视频讲解认真做笔记。 | Taking good notes over readings, PowerPoints, or video lectures. |
| LE7 | LE – emotional | 在设计学习中投入努力。 | Putting forth effort. |
| LE8 | LE – emotional | 设法让课程内容与自己的生活产生关联。 | Finding ways to make the course material relevant to my life. |
| LE9 | LE – emotional | 把课程所学应用到自己的生活中。 | Applying course material to my life. |
| LE10 | LE – emotional | 设法让课程对我而言变得有趣。 | Finding ways to make the course interesting to me. |
| LE11 | LE – emotional | 真心渴望学好课程内容。 | Really desiring to learn the material. |
| LE12 | LE – participation | 在与老师或同学的讨论交流中感到愉快。 | Having fun in online chats, discussions or via email with the instructor or other students. |
| LE13 | LE – participation | 积极参与小组讨论。 | Participating actively in small-group discussion forums. |
| LE14 | LE – participation | 帮助同学。 | Helping fellow students. |
| LE15 | LE – participation | 参与课程中的交流与讨论（线上/线下讨论、留言等）。 | Engaging in conversations online (chat, discussions, email). |
| LE16 | LE – participation | 经常在课程讨论中发言或发帖。 | Posting in the discussion forum regularly. |
| LE17 | LE – participation | 认识班上的其他同学。 | Getting to know other students in the class. |
| LE18 | LE – performance | 努力取得好成绩。 | Getting a good grade. |
| LE19 | LE – performance | 在课程的测验或作业评定中表现出色。 | Doing well on the tests/quizzes. |
| OR1 | AID – emotional | 没有AI的帮助时，我会感到不安或没有把握。 | I feel uneasy or insecure without the help of AI. |
| OR2 | AID – emotional | 用AI辅助设计学习让我感到平静、放松。 | Using AI to assist my studies makes me feel calm and relaxed. |
| OR3 | AID – emotional | 如果不能在设计学习中使用AI，我会感到焦虑和沮丧。 | I would feel anxious and frustrated if I couldn’t use AI for my studies. |
| OR4 | AID – emotional | 每当遇到设计难题，我的第一反应就是希望AI能帮我，否则就会紧张。 | Whenever I encounter a difficult problem, my first instinct is to hope AI can help me; otherwise, I feel tense. |
| OR5 | AID – emotional | 通过对话/提示与AI互动是一种愉快的体验，让我感到有支撑。 | Interacting with AI via chat prompts is a pleasant experience that makes me feel supported. |
| OR6 | AID – functional | 我几乎把所有设计任务都交给AI，因为它做得比我更快更好。 | I delegate almost all my academic tasks to AI because it performs them faster and better than I do. |
| OR7 | AID – functional | 我依赖AI的功能（如给出方案、提出修改建议）来提高设计学习效率。 | I rely on AI’s functions, including providing answers and writing suggestions, to improve my learning efficiency. |
| OR8 | AID – functional | 没有AI的协助，我已经难以完成复杂的设计任务。 | I can no longer manage complex academic tasks without the assistance of AI. |
| OR9 | AID – functional | 比起完全自己来，我更愿意把资料搜集和素材整理交给AI。 | I prefer to delegate information gathering and content organization to AI rather than doing it entirely myself. |
| OR10 | AID – functional | 在设计学习中，我过度依赖AI提供的现成方案或答案。 | In my studies, I excessively rely on the ready-made answers or solutions provided by AI. |
| OR11 | AID – functional | 没有AI的支持，我在设计学习中会感到力不从心。 | I feel inadequate in my studies without the support of AI. |
| OR12 | AID – cognitive | 我常常不先自己思考，就直接向AI要设计方案/答案。 | I often ask AI for answers directly without thinking for myself first. |
| OR13 | AID – cognitive | 使用AI之后，我独立分析和解决设计问题的能力下降了。 | After using AI, my ability to independently analyze and solve problems has declined. |
| OR14 | AID – cognitive | 面对设计上的挑战时，我会依赖AI，而不是先尝试自己想清楚。 | When faced with an academic challenge, I rely on AI instead of attempting to think it through on my own. |
| OR15 | AID – cognitive | 长期使用AI后，我越来越不愿独立思考，更愿意让AI来完成。 | After long-term use of AI, I have become less inclined to think independently, preferring AI to do the work. |
| OR16 | AID – cognitive | 我习惯让AI替我做判断和决定，而不是完全相信自己的判断。 | I am accustomed to letting AI make judgments and decisions for me rather than fully trusting my own judgment. |
| OR17 | AID – loss of control | 我曾试图减少使用AI，但从未坚持太久。 | I have tried to reduce my use of AI, but I have never succeeded for long. |
| OR18 | AID – loss of control | 一旦开始用AI，我常常很难停下来，即使已经花了很多时间。 | Once I start using AI, I often find it difficult to stop, even after spending a lot of time on it. |
| OR19 | AID – loss of control | 我知道不该太依赖AI，但我控制不住自己。 | I know I shouldn’t rely too much on AI, but I can’t control myself. |
| OR20 | AID – loss of control | 对于使用AI的频率和时长，我几乎无法控制。 | I have almost no control over the frequency and duration of my AI use. |
| OR21 | AID – loss of control | 我本可以不用AI完成任务，却仍忍不住要用。 | I am capable of completing tasks without AI, but I still cannot resist using it. |
| OR22 | AID – loss of control | 一段时间不用AI，我就会有强烈的使用冲动，有时甚至烦躁。 | If I don’t use AI for a period of time, I feel a strong urge to use it, sometimes even feeling irritable. |
| CSE1 | CSE | 我知道自己能够有效地解决即使很复杂的问题。 | I know I can efficiently solve even complicated problems. |
| CSE2 | CSE | 我信任自己的创造能力。 | I trust my creative abilities. |
| CSE3 | CSE | 我的想象力和独创性使我与同伴有所不同。 | My imagination and ingenuity distinguishes me from my friends. |
| CSE4 | CSE | 我曾多次证明自己能够应对困难的情境。 | Many times I have proved that I can cope with difficult situations. |
| CSE5 | CSE | 我确信自己能够处理需要创造性思考的问题。 | I am sure I can deal with problems requiring creative thinking. |
| CSE6 | CSE | 我擅长为问题提出独创性的解决方案。 | I am good at proposing original solutions to problems. |
| CPI1 | CPI | 我认为自己是一个有创造力的人。 | I think I am a creative person. |
| CPI2 | CPI | 创造力是“我是谁”的重要组成部分。 | My creativity is important for who I am. |
| CPI3 | CPI | 成为有创造力的人对我而言很重要。 | Being a creative person is important to me. |
| CPI4 | CPI | 创造力是我自我概念的重要组成部分。 | Creativity is an important part of myself. |
| CPI5 | CPI | 独创性是对我而言很重要的一种个人特质。 | Ingenuity is a characteristic that is important to me. |

**Note.** PL = self-reported prompt literacy; LE = learning engagement; AID = AI dependence (items OR1–OR22); CSE = creative self-efficacy; CPI = creative personal identity. All items used a 7-point scale. Sources: PL, Liu et al. (2025a), grouped by the interaction steps of Tour and Zadorozhnyy (2025); LE, Dixson (2015); AID, Wu et al. (2026); CSE and CPI, Karwowski (2012) and Karwowski et al. (2018). English items were translated and back-translated (see Measures).
